# Supplementary figures and images for: ASAR lncRNAs control DNA replication timing through interactions with multiple hnRNP/RNA binding proteins
Source: eLife. 2024 Jun 18;13:RP95898. doi: 10.7554/eLife.95898 (PMC11186638; doi:10.7554/eLife.95898)

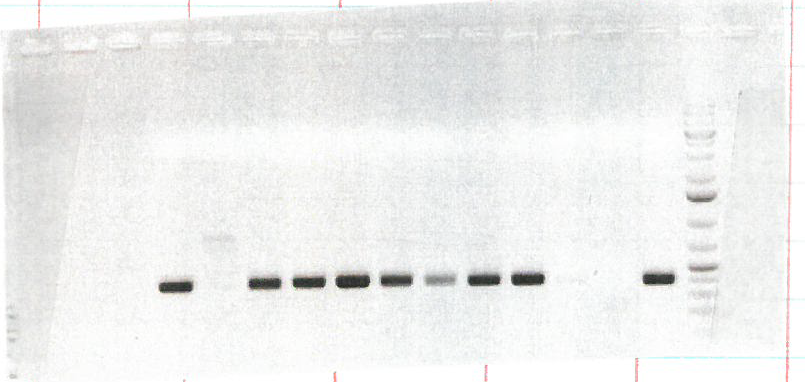

Supplement: Figure 1—figure supplement 1—source data 1. — Figure 1-figure supplement 1C, the original image file of the PCR products shown in panel C. Figure 1-figure supplement 1C (labels), the original image file for panel C with labels and regions of the image used are marked by the black boxes. Figure 1-figure supplement 1D, the original image file of the PCR products shown in panel D. Figure 1-figure supplement 1D (labels), the original image file for panel D with labels and regions of the image used are marked by the black boxes. [file elife-95898-fig1-figsupp1-data1.zip › Figure 1-figure supplement source file data/Figure 1-figure supplement 1D.tif]

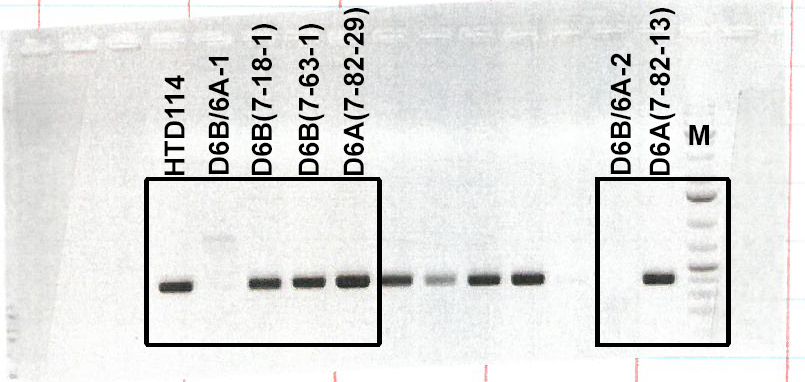

Supplement: Figure 1—figure supplement 1—source data 1. — Figure 1-figure supplement 1C, the original image file of the PCR products shown in panel C. Figure 1-figure supplement 1C (labels), the original image file for panel C with labels and regions of the image used are marked by the black boxes. Figure 1-figure supplement 1D, the original image file of the PCR products shown in panel D. Figure 1-figure supplement 1D (labels), the original image file for panel D with labels and regions of the image used are marked by the black boxes. [file elife-95898-fig1-figsupp1-data1.zip › Figure 1-figure supplement source file data/Figure 1-figure supplement 1D(labels).tif]

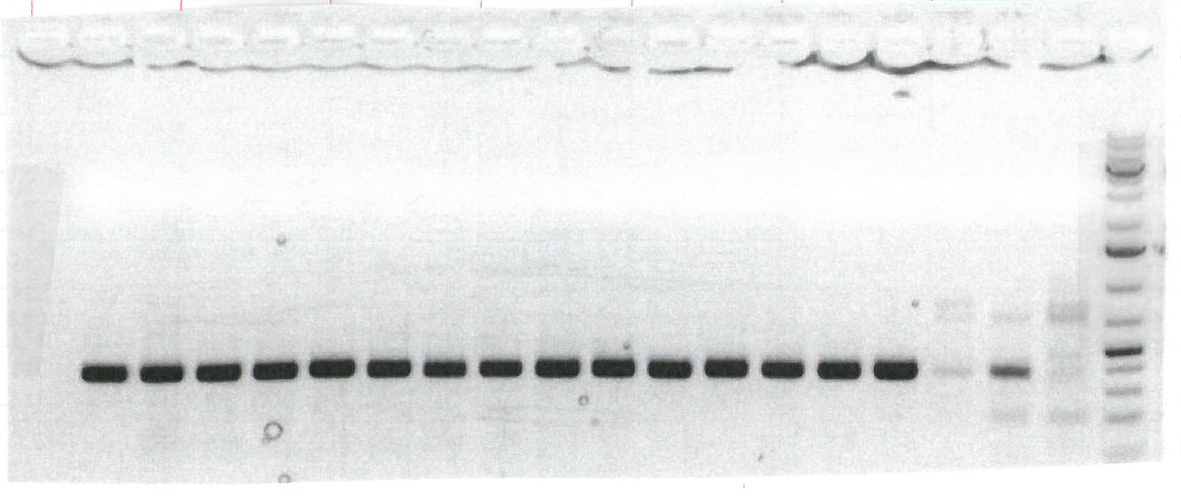

Supplement: Figure 1—figure supplement 1—source data 1. — Figure 1-figure supplement 1C, the original image file of the PCR products shown in panel C. Figure 1-figure supplement 1C (labels), the original image file for panel C with labels and regions of the image used are marked by the black boxes. Figure 1-figure supplement 1D, the original image file of the PCR products shown in panel D. Figure 1-figure supplement 1D (labels), the original image file for panel D with labels and regions of the image used are marked by the black boxes. [file elife-95898-fig1-figsupp1-data1.zip › Figure 1-figure supplement source file data/Figure 1-figure supplement 1C.tif]

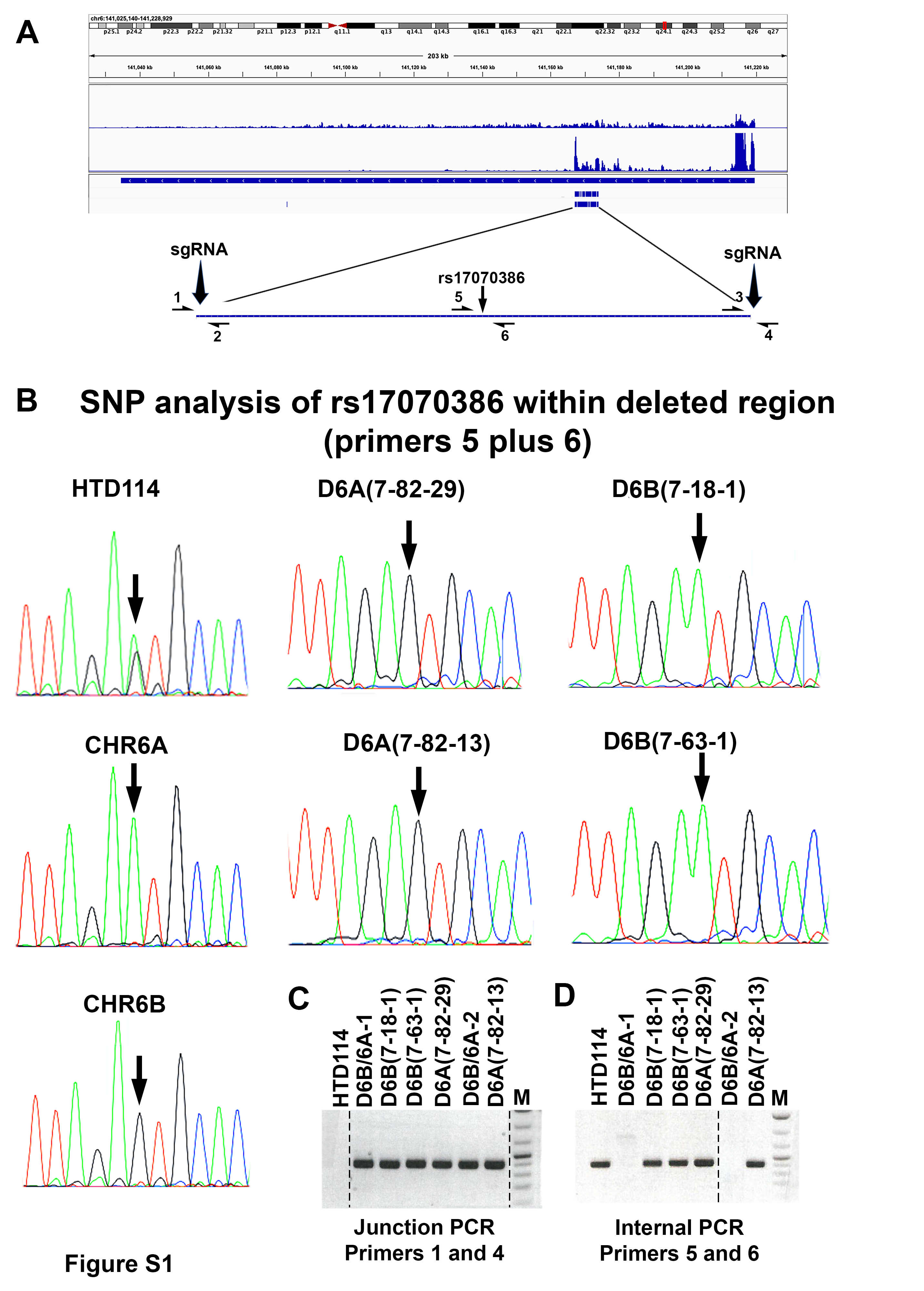

Supplement: Figure 1—figure supplement 1—source data 1. — Figure 1-figure supplement 1C, the original image file of the PCR products shown in panel C. Figure 1-figure supplement 1C (labels), the original image file for panel C with labels and regions of the image used are marked by the black boxes. Figure 1-figure supplement 1D, the original image file of the PCR products shown in panel D. Figure 1-figure supplement 1D (labels), the original image file for panel D with labels and regions of the image used are marked by the black boxes. [file elife-95898-fig1-figsupp1-data1.zip › Figure 1-figure supplement source file data/Figure 1-figure supplement 1.tif]

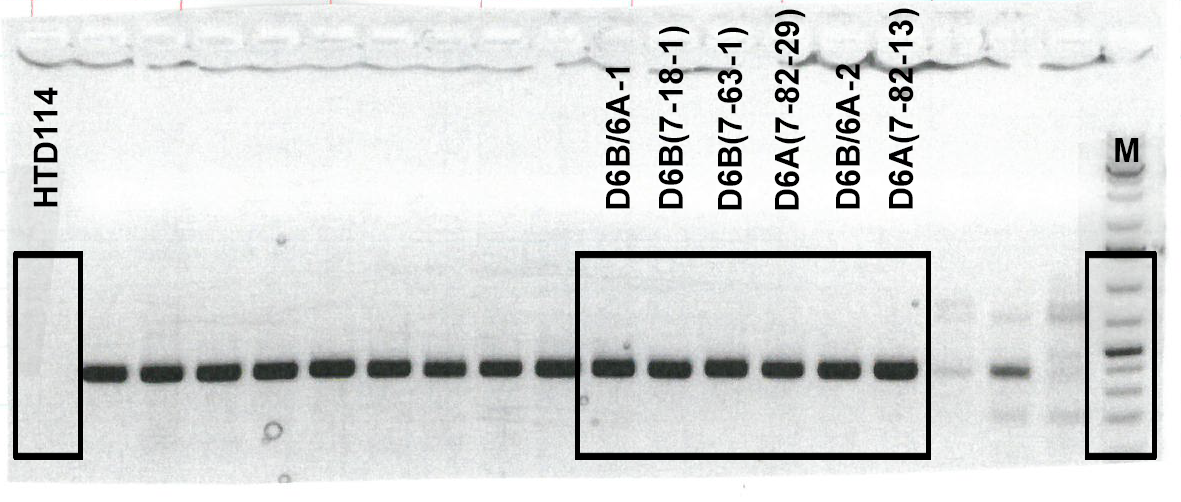

Supplement: Figure 1—figure supplement 1—source data 1. — Figure 1-figure supplement 1C, the original image file of the PCR products shown in panel C. Figure 1-figure supplement 1C (labels), the original image file for panel C with labels and regions of the image used are marked by the black boxes. Figure 1-figure supplement 1D, the original image file of the PCR products shown in panel D. Figure 1-figure supplement 1D (labels), the original image file for panel D with labels and regions of the image used are marked by the black boxes. [file elife-95898-fig1-figsupp1-data1.zip › Figure 1-figure supplement source file data/Figure 1-figure supplement 1C(labels).tif]

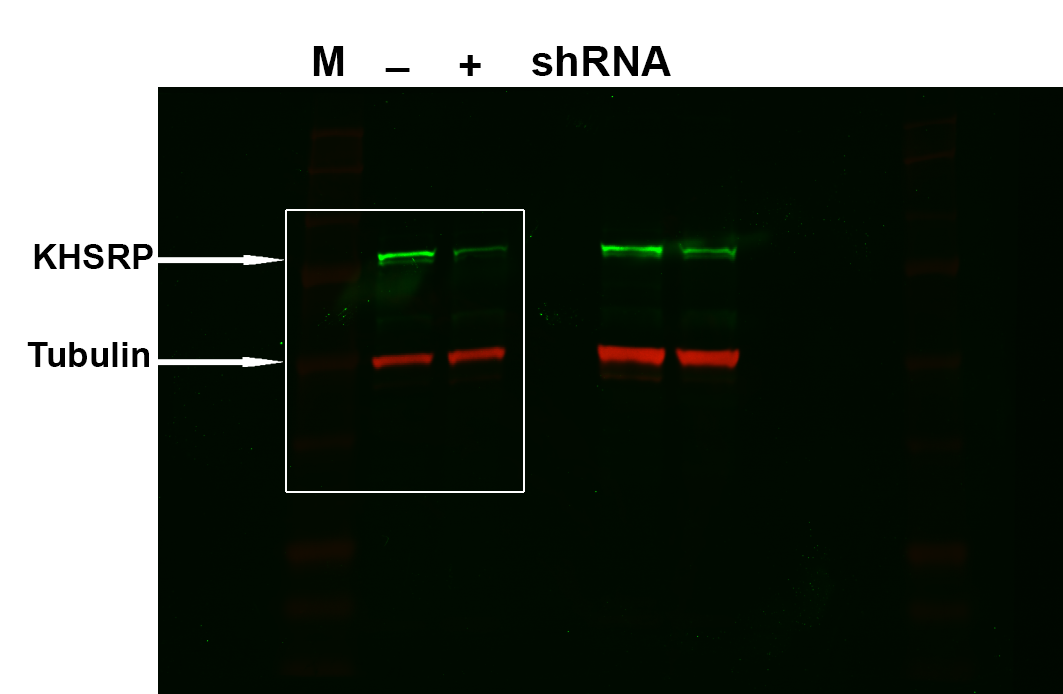

Supplement: Figure 3—figure supplement 1—source data 1. — Figure 3-figure supplement 1A, the original image file for the western blot with HNRNPU (green) and tubulin (red) antibodies on K562 cells with and without shRNA against HNRNPU. Figure 3-figure supplement 1A (labels), the same image as above showing labels for the location of HNRNPU and tubulin, and the region of the image used is highlighted in white. Molecular weight standards are shown in red. Figure 3-figure supplement 1B, the original image file for the western blot with HNRNPU (green) and tubulin (red) antibodies on HTD114 cells with and without shRNA against HNRNPU. Figure 3-figure supplement 1B (labels), the same image as above showing labels for the location of HNRNPU and tubulin, and the region of the image used is highlighted in white. Molecular weight standards are shown in red. Figure 3—figure supplement 1C, the original image file for the western blot with UCHL5 (green) and tubulin (red) antibodies on K562 cells with and without shRNA against UCHL5. Figure 3-figure supplement 1C (labels), the same image as above showing labels for the location of UCHL5 and tubulin, and the region of the image used is highlighted in white. Molecular weight standards are shown in red. Figure 3-figure supplement 1D, the original image file for the western blot with HNRNPC (green) and tubulin (red) antibodies on K562 cells with and without shRNA against HNRNPC. Figure 3-figure supplement 1C (labels), the same image as above showing labels for the location of HNRNPC and tubulin, and the region of the image used is highlighted in white. Molecular weight standards are shown in red. Figure 3—figure supplement 1E, the original image file for the western blot with HNRNPC (green) and tubulin (red) antibodies on HTD114 cells with and without shRNA against HNRNPC. Figure 3-figure supplement 1E (labels), the same image as above showing labels for the location of HNRNPC and tubulin, and the region of the image used is highlighted in white. Molecular weight standards are shown in red. [file elife-95898-fig3-figsupp1-data1.zip › Figure 3-figure supplement 1 source file data/Figure 3-figure supplement 1 J(labels).tif]

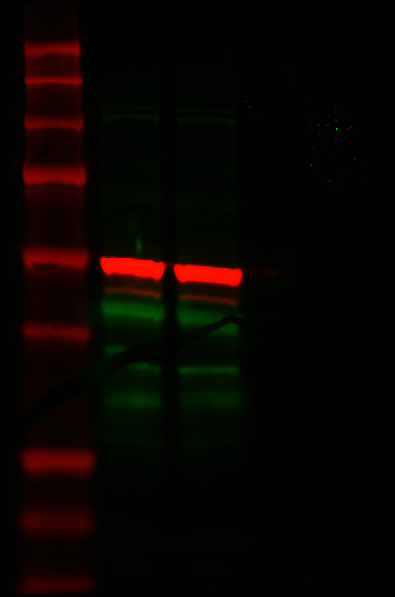

Supplement: Figure 3—figure supplement 1—source data 1. — Figure 3-figure supplement 1A, the original image file for the western blot with HNRNPU (green) and tubulin (red) antibodies on K562 cells with and without shRNA against HNRNPU. Figure 3-figure supplement 1A (labels), the same image as above showing labels for the location of HNRNPU and tubulin, and the region of the image used is highlighted in white. Molecular weight standards are shown in red. Figure 3-figure supplement 1B, the original image file for the western blot with HNRNPU (green) and tubulin (red) antibodies on HTD114 cells with and without shRNA against HNRNPU. Figure 3-figure supplement 1B (labels), the same image as above showing labels for the location of HNRNPU and tubulin, and the region of the image used is highlighted in white. Molecular weight standards are shown in red. Figure 3—figure supplement 1C, the original image file for the western blot with UCHL5 (green) and tubulin (red) antibodies on K562 cells with and without shRNA against UCHL5. Figure 3-figure supplement 1C (labels), the same image as above showing labels for the location of UCHL5 and tubulin, and the region of the image used is highlighted in white. Molecular weight standards are shown in red. Figure 3-figure supplement 1D, the original image file for the western blot with HNRNPC (green) and tubulin (red) antibodies on K562 cells with and without shRNA against HNRNPC. Figure 3-figure supplement 1C (labels), the same image as above showing labels for the location of HNRNPC and tubulin, and the region of the image used is highlighted in white. Molecular weight standards are shown in red. Figure 3—figure supplement 1E, the original image file for the western blot with HNRNPC (green) and tubulin (red) antibodies on HTD114 cells with and without shRNA against HNRNPC. Figure 3-figure supplement 1E (labels), the same image as above showing labels for the location of HNRNPC and tubulin, and the region of the image used is highlighted in white. Molecular weight standards are shown in red. [file elife-95898-fig3-figsupp1-data1.zip › Figure 3-figure supplement 1 source file data/Figure 3-figure supplement 1 P.tif]

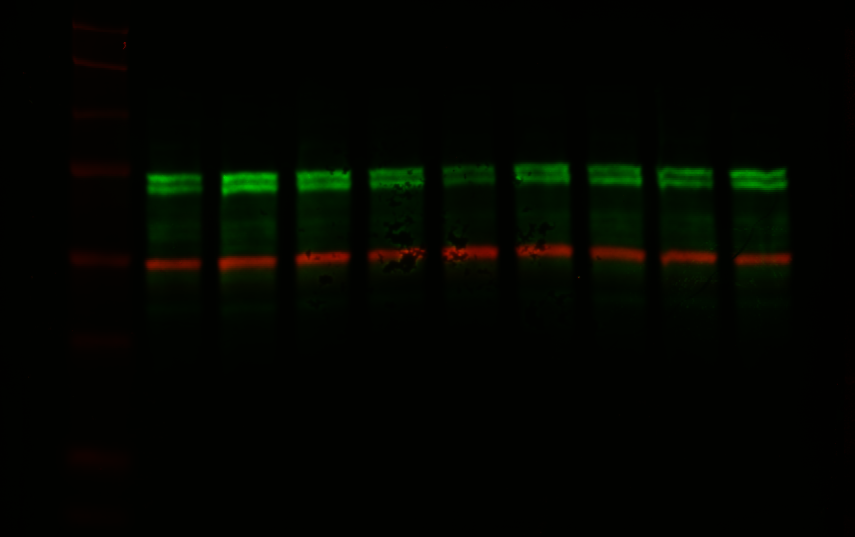

Supplement: Figure 3—figure supplement 1—source data 1. — Figure 3-figure supplement 1A, the original image file for the western blot with HNRNPU (green) and tubulin (red) antibodies on K562 cells with and without shRNA against HNRNPU. Figure 3-figure supplement 1A (labels), the same image as above showing labels for the location of HNRNPU and tubulin, and the region of the image used is highlighted in white. Molecular weight standards are shown in red. Figure 3-figure supplement 1B, the original image file for the western blot with HNRNPU (green) and tubulin (red) antibodies on HTD114 cells with and without shRNA against HNRNPU. Figure 3-figure supplement 1B (labels), the same image as above showing labels for the location of HNRNPU and tubulin, and the region of the image used is highlighted in white. Molecular weight standards are shown in red. Figure 3—figure supplement 1C, the original image file for the western blot with UCHL5 (green) and tubulin (red) antibodies on K562 cells with and without shRNA against UCHL5. Figure 3-figure supplement 1C (labels), the same image as above showing labels for the location of UCHL5 and tubulin, and the region of the image used is highlighted in white. Molecular weight standards are shown in red. Figure 3-figure supplement 1D, the original image file for the western blot with HNRNPC (green) and tubulin (red) antibodies on K562 cells with and without shRNA against HNRNPC. Figure 3-figure supplement 1C (labels), the same image as above showing labels for the location of HNRNPC and tubulin, and the region of the image used is highlighted in white. Molecular weight standards are shown in red. Figure 3—figure supplement 1E, the original image file for the western blot with HNRNPC (green) and tubulin (red) antibodies on HTD114 cells with and without shRNA against HNRNPC. Figure 3-figure supplement 1E (labels), the same image as above showing labels for the location of HNRNPC and tubulin, and the region of the image used is highlighted in white. Molecular weight standards are shown in red. [file elife-95898-fig3-figsupp1-data1.zip › Figure 3-figure supplement 1 source file data/Figure 3-figure supplement 1 G.tif]

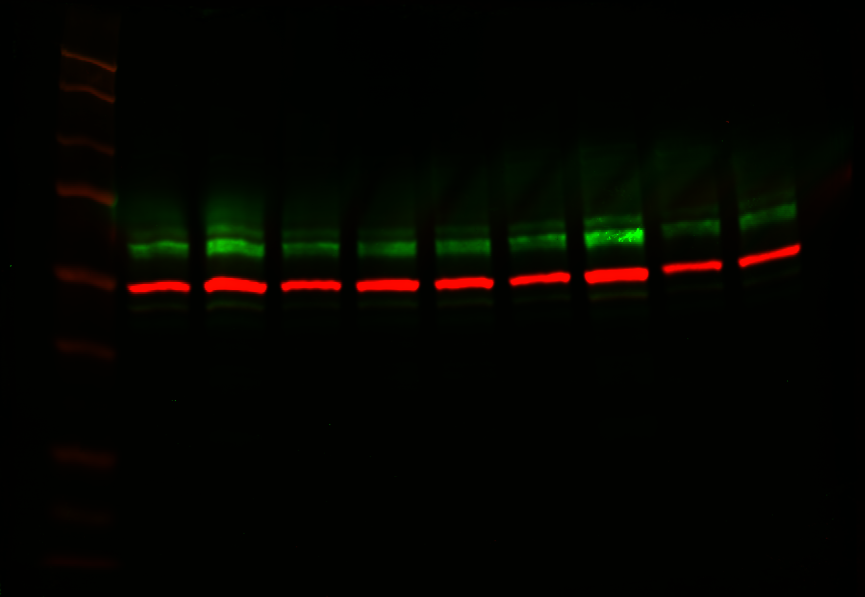

Supplement: Figure 3—figure supplement 1—source data 1. — Figure 3-figure supplement 1A, the original image file for the western blot with HNRNPU (green) and tubulin (red) antibodies on K562 cells with and without shRNA against HNRNPU. Figure 3-figure supplement 1A (labels), the same image as above showing labels for the location of HNRNPU and tubulin, and the region of the image used is highlighted in white. Molecular weight standards are shown in red. Figure 3-figure supplement 1B, the original image file for the western blot with HNRNPU (green) and tubulin (red) antibodies on HTD114 cells with and without shRNA against HNRNPU. Figure 3-figure supplement 1B (labels), the same image as above showing labels for the location of HNRNPU and tubulin, and the region of the image used is highlighted in white. Molecular weight standards are shown in red. Figure 3—figure supplement 1C, the original image file for the western blot with UCHL5 (green) and tubulin (red) antibodies on K562 cells with and without shRNA against UCHL5. Figure 3-figure supplement 1C (labels), the same image as above showing labels for the location of UCHL5 and tubulin, and the region of the image used is highlighted in white. Molecular weight standards are shown in red. Figure 3-figure supplement 1D, the original image file for the western blot with HNRNPC (green) and tubulin (red) antibodies on K562 cells with and without shRNA against HNRNPC. Figure 3-figure supplement 1C (labels), the same image as above showing labels for the location of HNRNPC and tubulin, and the region of the image used is highlighted in white. Molecular weight standards are shown in red. Figure 3—figure supplement 1E, the original image file for the western blot with HNRNPC (green) and tubulin (red) antibodies on HTD114 cells with and without shRNA against HNRNPC. Figure 3-figure supplement 1E (labels), the same image as above showing labels for the location of HNRNPC and tubulin, and the region of the image used is highlighted in white. Molecular weight standards are shown in red. [file elife-95898-fig3-figsupp1-data1.zip › Figure 3-figure supplement 1 source file data/Figure 3-figure supplement 1 F.tif]

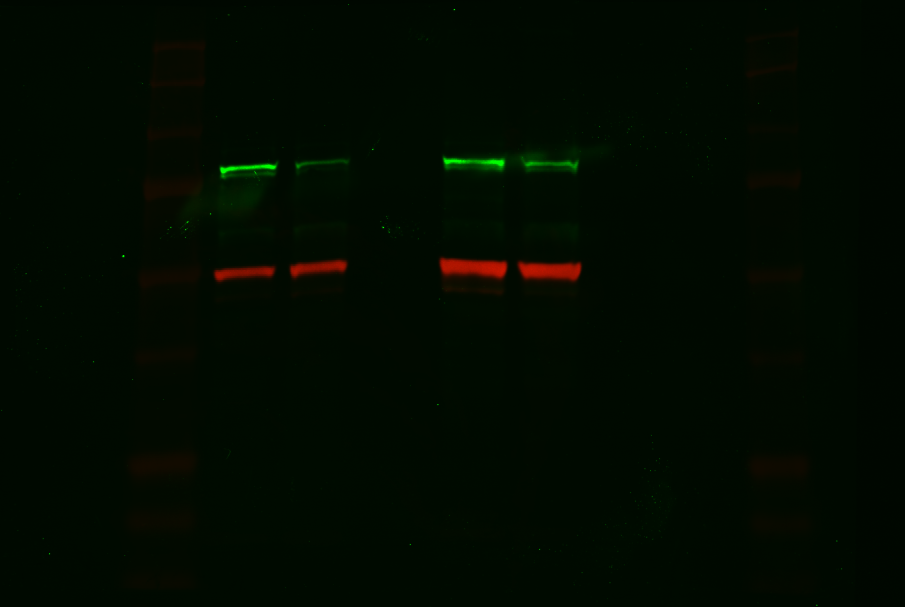

Supplement: Figure 3—figure supplement 1—source data 1. — Figure 3-figure supplement 1A, the original image file for the western blot with HNRNPU (green) and tubulin (red) antibodies on K562 cells with and without shRNA against HNRNPU. Figure 3-figure supplement 1A (labels), the same image as above showing labels for the location of HNRNPU and tubulin, and the region of the image used is highlighted in white. Molecular weight standards are shown in red. Figure 3-figure supplement 1B, the original image file for the western blot with HNRNPU (green) and tubulin (red) antibodies on HTD114 cells with and without shRNA against HNRNPU. Figure 3-figure supplement 1B (labels), the same image as above showing labels for the location of HNRNPU and tubulin, and the region of the image used is highlighted in white. Molecular weight standards are shown in red. Figure 3—figure supplement 1C, the original image file for the western blot with UCHL5 (green) and tubulin (red) antibodies on K562 cells with and without shRNA against UCHL5. Figure 3-figure supplement 1C (labels), the same image as above showing labels for the location of UCHL5 and tubulin, and the region of the image used is highlighted in white. Molecular weight standards are shown in red. Figure 3-figure supplement 1D, the original image file for the western blot with HNRNPC (green) and tubulin (red) antibodies on K562 cells with and without shRNA against HNRNPC. Figure 3-figure supplement 1C (labels), the same image as above showing labels for the location of HNRNPC and tubulin, and the region of the image used is highlighted in white. Molecular weight standards are shown in red. Figure 3—figure supplement 1E, the original image file for the western blot with HNRNPC (green) and tubulin (red) antibodies on HTD114 cells with and without shRNA against HNRNPC. Figure 3-figure supplement 1E (labels), the same image as above showing labels for the location of HNRNPC and tubulin, and the region of the image used is highlighted in white. Molecular weight standards are shown in red. [file elife-95898-fig3-figsupp1-data1.zip › Figure 3-figure supplement 1 source file data/Figure 3-figure supplement 1 J&K.tif]

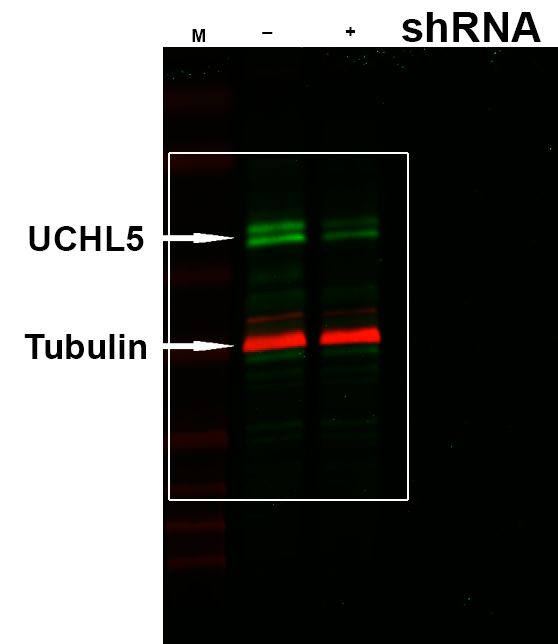

Supplement: Figure 3—figure supplement 1—source data 1. — Figure 3-figure supplement 1A, the original image file for the western blot with HNRNPU (green) and tubulin (red) antibodies on K562 cells with and without shRNA against HNRNPU. Figure 3-figure supplement 1A (labels), the same image as above showing labels for the location of HNRNPU and tubulin, and the region of the image used is highlighted in white. Molecular weight standards are shown in red. Figure 3-figure supplement 1B, the original image file for the western blot with HNRNPU (green) and tubulin (red) antibodies on HTD114 cells with and without shRNA against HNRNPU. Figure 3-figure supplement 1B (labels), the same image as above showing labels for the location of HNRNPU and tubulin, and the region of the image used is highlighted in white. Molecular weight standards are shown in red. Figure 3—figure supplement 1C, the original image file for the western blot with UCHL5 (green) and tubulin (red) antibodies on K562 cells with and without shRNA against UCHL5. Figure 3-figure supplement 1C (labels), the same image as above showing labels for the location of UCHL5 and tubulin, and the region of the image used is highlighted in white. Molecular weight standards are shown in red. Figure 3-figure supplement 1D, the original image file for the western blot with HNRNPC (green) and tubulin (red) antibodies on K562 cells with and without shRNA against HNRNPC. Figure 3-figure supplement 1C (labels), the same image as above showing labels for the location of HNRNPC and tubulin, and the region of the image used is highlighted in white. Molecular weight standards are shown in red. Figure 3—figure supplement 1E, the original image file for the western blot with HNRNPC (green) and tubulin (red) antibodies on HTD114 cells with and without shRNA against HNRNPC. Figure 3-figure supplement 1E (labels), the same image as above showing labels for the location of HNRNPC and tubulin, and the region of the image used is highlighted in white. Molecular weight standards are shown in red. [file elife-95898-fig3-figsupp1-data1.zip › Figure 3-figure supplement 1 source file data/Figure 3-figure supplement 1 C(labels).tif]

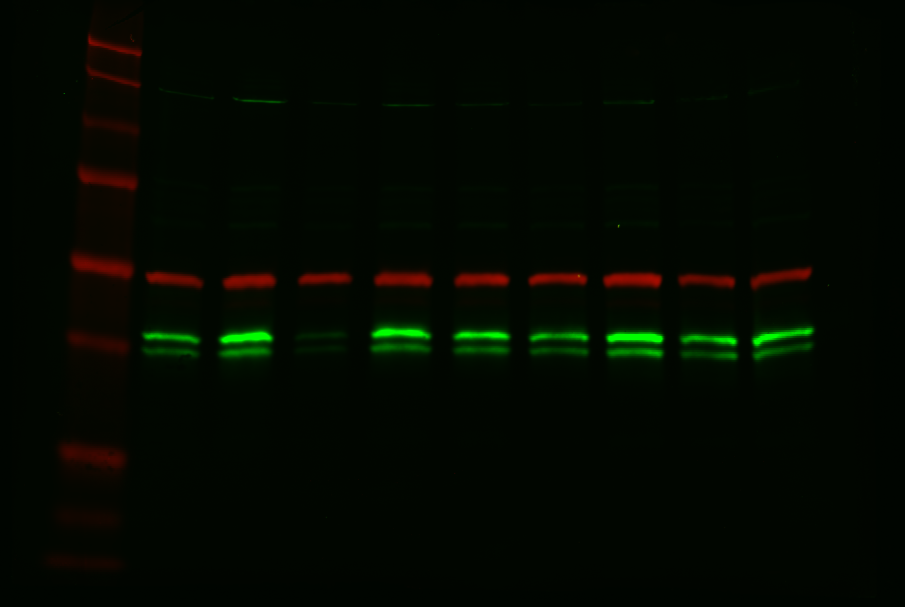

Supplement: Figure 3—figure supplement 1—source data 1. — Figure 3-figure supplement 1A, the original image file for the western blot with HNRNPU (green) and tubulin (red) antibodies on K562 cells with and without shRNA against HNRNPU. Figure 3-figure supplement 1A (labels), the same image as above showing labels for the location of HNRNPU and tubulin, and the region of the image used is highlighted in white. Molecular weight standards are shown in red. Figure 3-figure supplement 1B, the original image file for the western blot with HNRNPU (green) and tubulin (red) antibodies on HTD114 cells with and without shRNA against HNRNPU. Figure 3-figure supplement 1B (labels), the same image as above showing labels for the location of HNRNPU and tubulin, and the region of the image used is highlighted in white. Molecular weight standards are shown in red. Figure 3—figure supplement 1C, the original image file for the western blot with UCHL5 (green) and tubulin (red) antibodies on K562 cells with and without shRNA against UCHL5. Figure 3-figure supplement 1C (labels), the same image as above showing labels for the location of UCHL5 and tubulin, and the region of the image used is highlighted in white. Molecular weight standards are shown in red. Figure 3-figure supplement 1D, the original image file for the western blot with HNRNPC (green) and tubulin (red) antibodies on K562 cells with and without shRNA against HNRNPC. Figure 3-figure supplement 1C (labels), the same image as above showing labels for the location of HNRNPC and tubulin, and the region of the image used is highlighted in white. Molecular weight standards are shown in red. Figure 3—figure supplement 1E, the original image file for the western blot with HNRNPC (green) and tubulin (red) antibodies on HTD114 cells with and without shRNA against HNRNPC. Figure 3-figure supplement 1E (labels), the same image as above showing labels for the location of HNRNPC and tubulin, and the region of the image used is highlighted in white. Molecular weight standards are shown in red. [file elife-95898-fig3-figsupp1-data1.zip › Figure 3-figure supplement 1 source file data/Figure 3-figure supplement 1 D.tif]

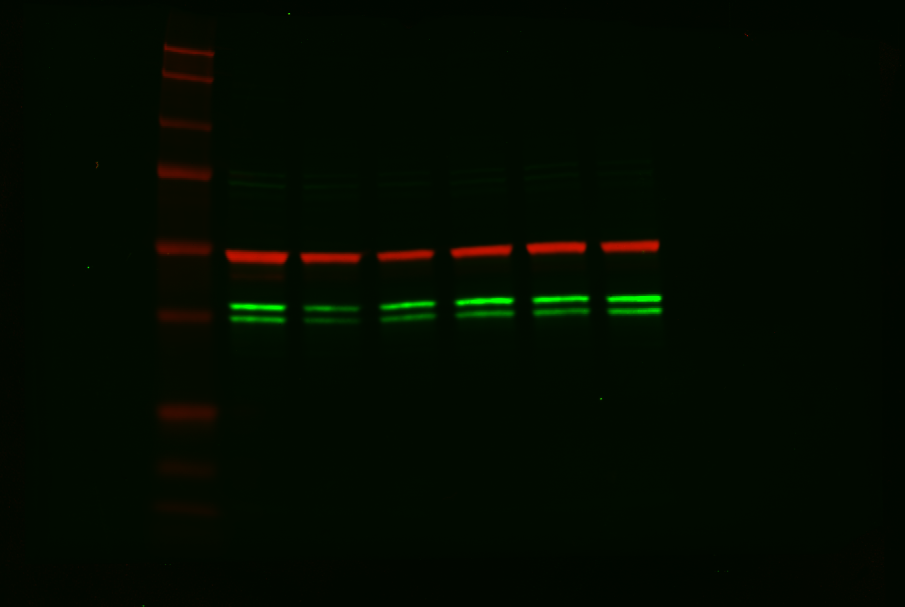

Supplement: Figure 3—figure supplement 1—source data 1. — Figure 3-figure supplement 1A, the original image file for the western blot with HNRNPU (green) and tubulin (red) antibodies on K562 cells with and without shRNA against HNRNPU. Figure 3-figure supplement 1A (labels), the same image as above showing labels for the location of HNRNPU and tubulin, and the region of the image used is highlighted in white. Molecular weight standards are shown in red. Figure 3-figure supplement 1B, the original image file for the western blot with HNRNPU (green) and tubulin (red) antibodies on HTD114 cells with and without shRNA against HNRNPU. Figure 3-figure supplement 1B (labels), the same image as above showing labels for the location of HNRNPU and tubulin, and the region of the image used is highlighted in white. Molecular weight standards are shown in red. Figure 3—figure supplement 1C, the original image file for the western blot with UCHL5 (green) and tubulin (red) antibodies on K562 cells with and without shRNA against UCHL5. Figure 3-figure supplement 1C (labels), the same image as above showing labels for the location of UCHL5 and tubulin, and the region of the image used is highlighted in white. Molecular weight standards are shown in red. Figure 3-figure supplement 1D, the original image file for the western blot with HNRNPC (green) and tubulin (red) antibodies on K562 cells with and without shRNA against HNRNPC. Figure 3-figure supplement 1C (labels), the same image as above showing labels for the location of HNRNPC and tubulin, and the region of the image used is highlighted in white. Molecular weight standards are shown in red. Figure 3—figure supplement 1E, the original image file for the western blot with HNRNPC (green) and tubulin (red) antibodies on HTD114 cells with and without shRNA against HNRNPC. Figure 3-figure supplement 1E (labels), the same image as above showing labels for the location of HNRNPC and tubulin, and the region of the image used is highlighted in white. Molecular weight standards are shown in red. [file elife-95898-fig3-figsupp1-data1.zip › Figure 3-figure supplement 1 source file data/Figure 3-figure supplement 1 E.tif]

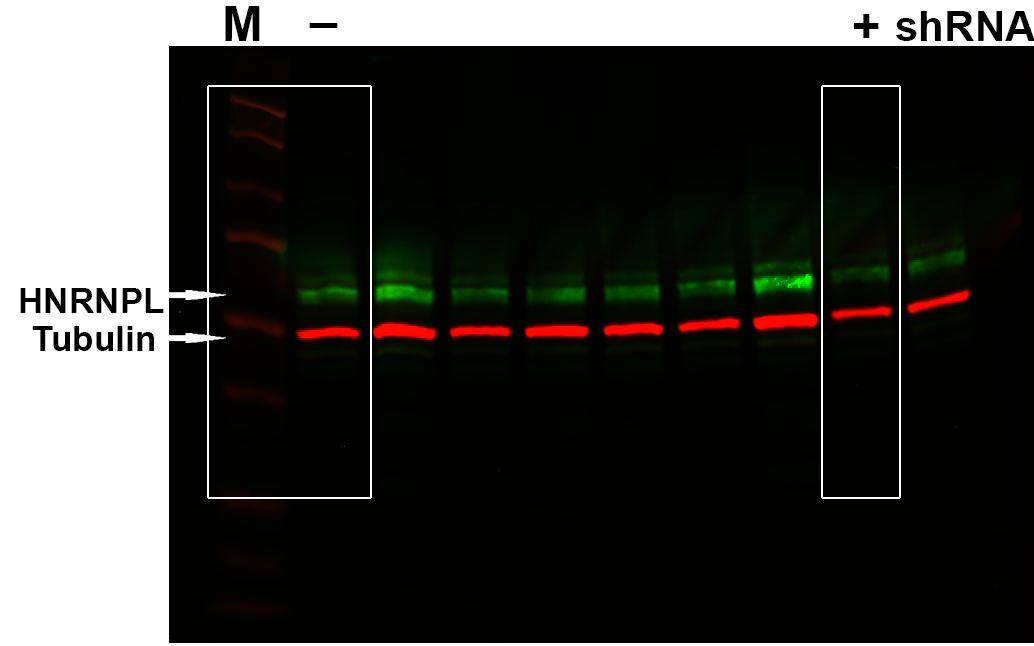

Supplement: Figure 3—figure supplement 1—source data 1. — Figure 3-figure supplement 1A, the original image file for the western blot with HNRNPU (green) and tubulin (red) antibodies on K562 cells with and without shRNA against HNRNPU. Figure 3-figure supplement 1A (labels), the same image as above showing labels for the location of HNRNPU and tubulin, and the region of the image used is highlighted in white. Molecular weight standards are shown in red. Figure 3-figure supplement 1B, the original image file for the western blot with HNRNPU (green) and tubulin (red) antibodies on HTD114 cells with and without shRNA against HNRNPU. Figure 3-figure supplement 1B (labels), the same image as above showing labels for the location of HNRNPU and tubulin, and the region of the image used is highlighted in white. Molecular weight standards are shown in red. Figure 3—figure supplement 1C, the original image file for the western blot with UCHL5 (green) and tubulin (red) antibodies on K562 cells with and without shRNA against UCHL5. Figure 3-figure supplement 1C (labels), the same image as above showing labels for the location of UCHL5 and tubulin, and the region of the image used is highlighted in white. Molecular weight standards are shown in red. Figure 3-figure supplement 1D, the original image file for the western blot with HNRNPC (green) and tubulin (red) antibodies on K562 cells with and without shRNA against HNRNPC. Figure 3-figure supplement 1C (labels), the same image as above showing labels for the location of HNRNPC and tubulin, and the region of the image used is highlighted in white. Molecular weight standards are shown in red. Figure 3—figure supplement 1E, the original image file for the western blot with HNRNPC (green) and tubulin (red) antibodies on HTD114 cells with and without shRNA against HNRNPC. Figure 3-figure supplement 1E (labels), the same image as above showing labels for the location of HNRNPC and tubulin, and the region of the image used is highlighted in white. Molecular weight standards are shown in red. [file elife-95898-fig3-figsupp1-data1.zip › Figure 3-figure supplement 1 source file data/Figure 3-figure supplement 1 F(labels).tif]

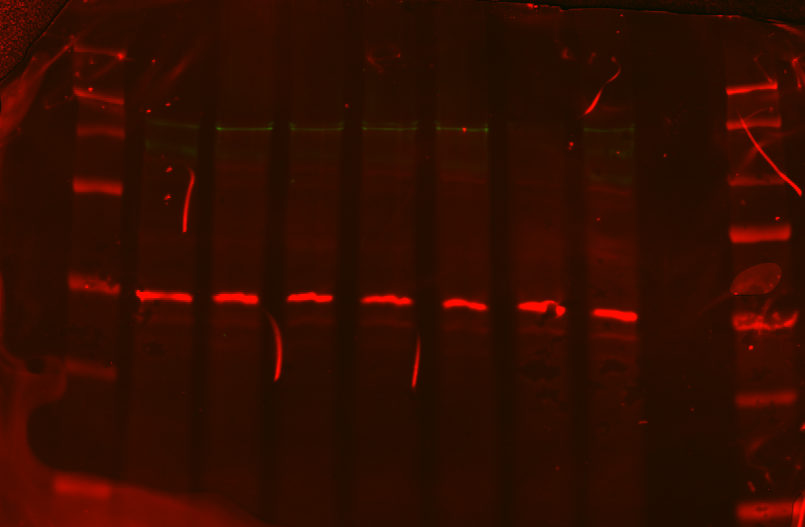

Supplement: Figure 3—figure supplement 1—source data 1. — Figure 3-figure supplement 1A, the original image file for the western blot with HNRNPU (green) and tubulin (red) antibodies on K562 cells with and without shRNA against HNRNPU. Figure 3-figure supplement 1A (labels), the same image as above showing labels for the location of HNRNPU and tubulin, and the region of the image used is highlighted in white. Molecular weight standards are shown in red. Figure 3-figure supplement 1B, the original image file for the western blot with HNRNPU (green) and tubulin (red) antibodies on HTD114 cells with and without shRNA against HNRNPU. Figure 3-figure supplement 1B (labels), the same image as above showing labels for the location of HNRNPU and tubulin, and the region of the image used is highlighted in white. Molecular weight standards are shown in red. Figure 3—figure supplement 1C, the original image file for the western blot with UCHL5 (green) and tubulin (red) antibodies on K562 cells with and without shRNA against UCHL5. Figure 3-figure supplement 1C (labels), the same image as above showing labels for the location of UCHL5 and tubulin, and the region of the image used is highlighted in white. Molecular weight standards are shown in red. Figure 3-figure supplement 1D, the original image file for the western blot with HNRNPC (green) and tubulin (red) antibodies on K562 cells with and without shRNA against HNRNPC. Figure 3-figure supplement 1C (labels), the same image as above showing labels for the location of HNRNPC and tubulin, and the region of the image used is highlighted in white. Molecular weight standards are shown in red. Figure 3—figure supplement 1E, the original image file for the western blot with HNRNPC (green) and tubulin (red) antibodies on HTD114 cells with and without shRNA against HNRNPC. Figure 3-figure supplement 1E (labels), the same image as above showing labels for the location of HNRNPC and tubulin, and the region of the image used is highlighted in white. Molecular weight standards are shown in red. [file elife-95898-fig3-figsupp1-data1.zip › Figure 3-figure supplement 1 source file data/Figure 3-figure supplement 1 A.tif]

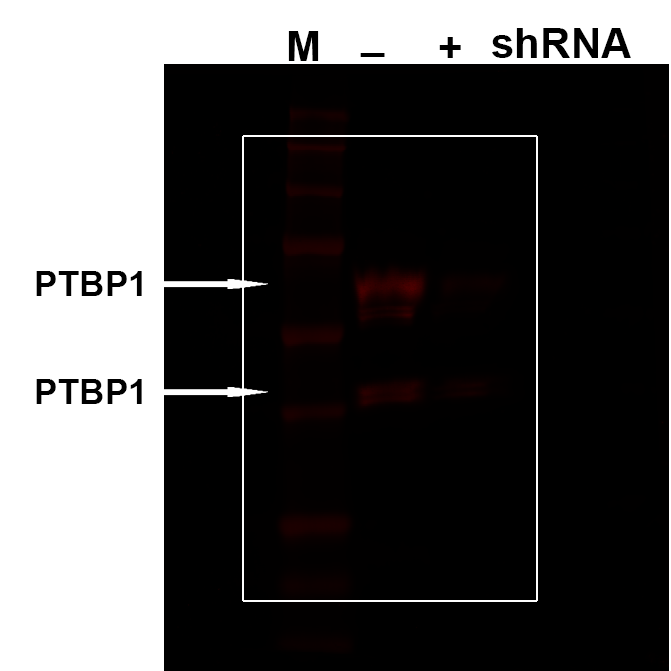

Supplement: Figure 3—figure supplement 1—source data 1. — Figure 3-figure supplement 1A, the original image file for the western blot with HNRNPU (green) and tubulin (red) antibodies on K562 cells with and without shRNA against HNRNPU. Figure 3-figure supplement 1A (labels), the same image as above showing labels for the location of HNRNPU and tubulin, and the region of the image used is highlighted in white. Molecular weight standards are shown in red. Figure 3-figure supplement 1B, the original image file for the western blot with HNRNPU (green) and tubulin (red) antibodies on HTD114 cells with and without shRNA against HNRNPU. Figure 3-figure supplement 1B (labels), the same image as above showing labels for the location of HNRNPU and tubulin, and the region of the image used is highlighted in white. Molecular weight standards are shown in red. Figure 3—figure supplement 1C, the original image file for the western blot with UCHL5 (green) and tubulin (red) antibodies on K562 cells with and without shRNA against UCHL5. Figure 3-figure supplement 1C (labels), the same image as above showing labels for the location of UCHL5 and tubulin, and the region of the image used is highlighted in white. Molecular weight standards are shown in red. Figure 3-figure supplement 1D, the original image file for the western blot with HNRNPC (green) and tubulin (red) antibodies on K562 cells with and without shRNA against HNRNPC. Figure 3-figure supplement 1C (labels), the same image as above showing labels for the location of HNRNPC and tubulin, and the region of the image used is highlighted in white. Molecular weight standards are shown in red. Figure 3—figure supplement 1E, the original image file for the western blot with HNRNPC (green) and tubulin (red) antibodies on HTD114 cells with and without shRNA against HNRNPC. Figure 3-figure supplement 1E (labels), the same image as above showing labels for the location of HNRNPC and tubulin, and the region of the image used is highlighted in white. Molecular weight standards are shown in red. [file elife-95898-fig3-figsupp1-data1.zip › Figure 3-figure supplement 1 source file data/Figure 3-figure supplement 1 O(Labels).tif]

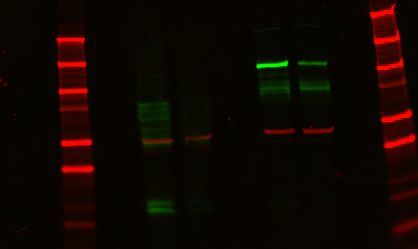

Supplement: Figure 3—figure supplement 1—source data 1. — Figure 3-figure supplement 1A, the original image file for the western blot with HNRNPU (green) and tubulin (red) antibodies on K562 cells with and without shRNA against HNRNPU. Figure 3-figure supplement 1A (labels), the same image as above showing labels for the location of HNRNPU and tubulin, and the region of the image used is highlighted in white. Molecular weight standards are shown in red. Figure 3-figure supplement 1B, the original image file for the western blot with HNRNPU (green) and tubulin (red) antibodies on HTD114 cells with and without shRNA against HNRNPU. Figure 3-figure supplement 1B (labels), the same image as above showing labels for the location of HNRNPU and tubulin, and the region of the image used is highlighted in white. Molecular weight standards are shown in red. Figure 3—figure supplement 1C, the original image file for the western blot with UCHL5 (green) and tubulin (red) antibodies on K562 cells with and without shRNA against UCHL5. Figure 3-figure supplement 1C (labels), the same image as above showing labels for the location of UCHL5 and tubulin, and the region of the image used is highlighted in white. Molecular weight standards are shown in red. Figure 3-figure supplement 1D, the original image file for the western blot with HNRNPC (green) and tubulin (red) antibodies on K562 cells with and without shRNA against HNRNPC. Figure 3-figure supplement 1C (labels), the same image as above showing labels for the location of HNRNPC and tubulin, and the region of the image used is highlighted in white. Molecular weight standards are shown in red. Figure 3—figure supplement 1E, the original image file for the western blot with HNRNPC (green) and tubulin (red) antibodies on HTD114 cells with and without shRNA against HNRNPC. Figure 3-figure supplement 1E (labels), the same image as above showing labels for the location of HNRNPC and tubulin, and the region of the image used is highlighted in white. Molecular weight standards are shown in red. [file elife-95898-fig3-figsupp1-data1.zip › Figure 3-figure supplement 1 source file data/Figure 3-figure supplement 1 B.tif]

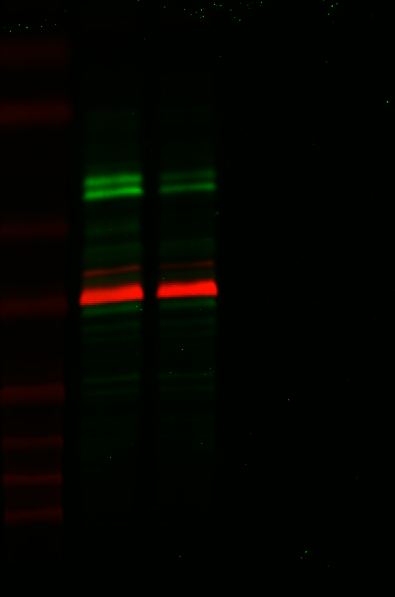

Supplement: Figure 3—figure supplement 1—source data 1. — Figure 3-figure supplement 1A, the original image file for the western blot with HNRNPU (green) and tubulin (red) antibodies on K562 cells with and without shRNA against HNRNPU. Figure 3-figure supplement 1A (labels), the same image as above showing labels for the location of HNRNPU and tubulin, and the region of the image used is highlighted in white. Molecular weight standards are shown in red. Figure 3-figure supplement 1B, the original image file for the western blot with HNRNPU (green) and tubulin (red) antibodies on HTD114 cells with and without shRNA against HNRNPU. Figure 3-figure supplement 1B (labels), the same image as above showing labels for the location of HNRNPU and tubulin, and the region of the image used is highlighted in white. Molecular weight standards are shown in red. Figure 3—figure supplement 1C, the original image file for the western blot with UCHL5 (green) and tubulin (red) antibodies on K562 cells with and without shRNA against UCHL5. Figure 3-figure supplement 1C (labels), the same image as above showing labels for the location of UCHL5 and tubulin, and the region of the image used is highlighted in white. Molecular weight standards are shown in red. Figure 3-figure supplement 1D, the original image file for the western blot with HNRNPC (green) and tubulin (red) antibodies on K562 cells with and without shRNA against HNRNPC. Figure 3-figure supplement 1C (labels), the same image as above showing labels for the location of HNRNPC and tubulin, and the region of the image used is highlighted in white. Molecular weight standards are shown in red. Figure 3—figure supplement 1E, the original image file for the western blot with HNRNPC (green) and tubulin (red) antibodies on HTD114 cells with and without shRNA against HNRNPC. Figure 3-figure supplement 1E (labels), the same image as above showing labels for the location of HNRNPC and tubulin, and the region of the image used is highlighted in white. Molecular weight standards are shown in red. [file elife-95898-fig3-figsupp1-data1.zip › Figure 3-figure supplement 1 source file data/Figure 3-figure supplement 1 C.tif]

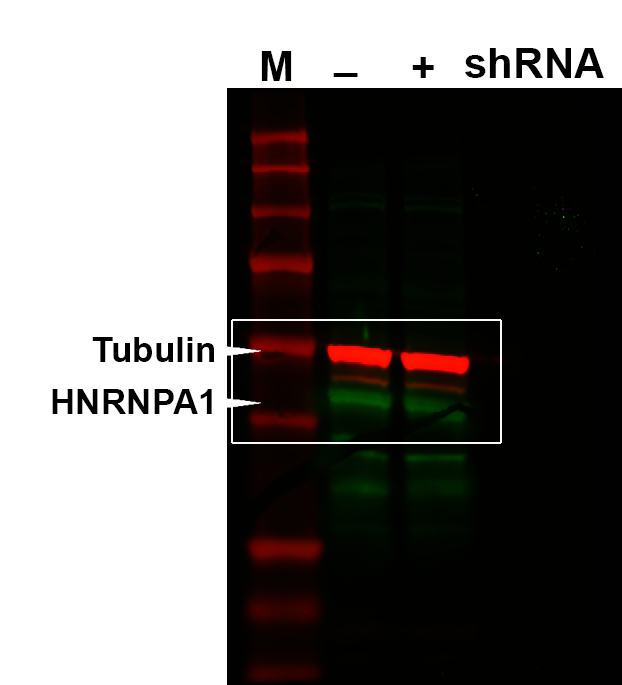

Supplement: Figure 3—figure supplement 1—source data 1. — Figure 3-figure supplement 1A, the original image file for the western blot with HNRNPU (green) and tubulin (red) antibodies on K562 cells with and without shRNA against HNRNPU. Figure 3-figure supplement 1A (labels), the same image as above showing labels for the location of HNRNPU and tubulin, and the region of the image used is highlighted in white. Molecular weight standards are shown in red. Figure 3-figure supplement 1B, the original image file for the western blot with HNRNPU (green) and tubulin (red) antibodies on HTD114 cells with and without shRNA against HNRNPU. Figure 3-figure supplement 1B (labels), the same image as above showing labels for the location of HNRNPU and tubulin, and the region of the image used is highlighted in white. Molecular weight standards are shown in red. Figure 3—figure supplement 1C, the original image file for the western blot with UCHL5 (green) and tubulin (red) antibodies on K562 cells with and without shRNA against UCHL5. Figure 3-figure supplement 1C (labels), the same image as above showing labels for the location of UCHL5 and tubulin, and the region of the image used is highlighted in white. Molecular weight standards are shown in red. Figure 3-figure supplement 1D, the original image file for the western blot with HNRNPC (green) and tubulin (red) antibodies on K562 cells with and without shRNA against HNRNPC. Figure 3-figure supplement 1C (labels), the same image as above showing labels for the location of HNRNPC and tubulin, and the region of the image used is highlighted in white. Molecular weight standards are shown in red. Figure 3—figure supplement 1E, the original image file for the western blot with HNRNPC (green) and tubulin (red) antibodies on HTD114 cells with and without shRNA against HNRNPC. Figure 3-figure supplement 1E (labels), the same image as above showing labels for the location of HNRNPC and tubulin, and the region of the image used is highlighted in white. Molecular weight standards are shown in red. [file elife-95898-fig3-figsupp1-data1.zip › Figure 3-figure supplement 1 source file data/Figure 3-figure supplement 1 P(labels).tif]

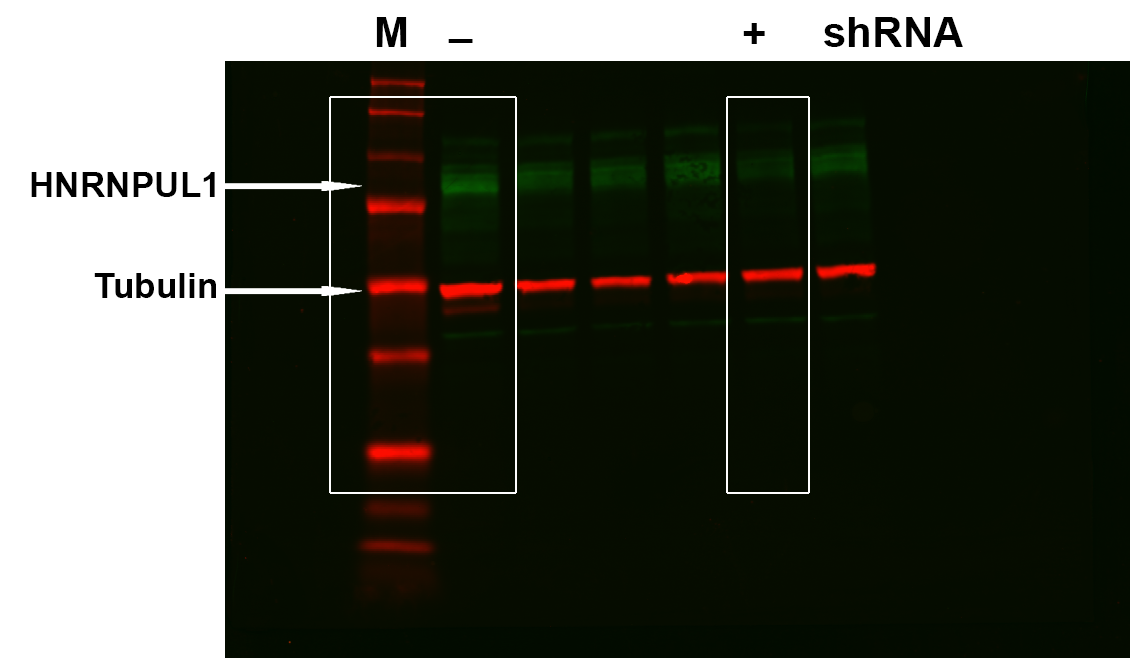

Supplement: Figure 3—figure supplement 1—source data 1. — Figure 3-figure supplement 1A, the original image file for the western blot with HNRNPU (green) and tubulin (red) antibodies on K562 cells with and without shRNA against HNRNPU. Figure 3-figure supplement 1A (labels), the same image as above showing labels for the location of HNRNPU and tubulin, and the region of the image used is highlighted in white. Molecular weight standards are shown in red. Figure 3-figure supplement 1B, the original image file for the western blot with HNRNPU (green) and tubulin (red) antibodies on HTD114 cells with and without shRNA against HNRNPU. Figure 3-figure supplement 1B (labels), the same image as above showing labels for the location of HNRNPU and tubulin, and the region of the image used is highlighted in white. Molecular weight standards are shown in red. Figure 3—figure supplement 1C, the original image file for the western blot with UCHL5 (green) and tubulin (red) antibodies on K562 cells with and without shRNA against UCHL5. Figure 3-figure supplement 1C (labels), the same image as above showing labels for the location of UCHL5 and tubulin, and the region of the image used is highlighted in white. Molecular weight standards are shown in red. Figure 3-figure supplement 1D, the original image file for the western blot with HNRNPC (green) and tubulin (red) antibodies on K562 cells with and without shRNA against HNRNPC. Figure 3-figure supplement 1C (labels), the same image as above showing labels for the location of HNRNPC and tubulin, and the region of the image used is highlighted in white. Molecular weight standards are shown in red. Figure 3—figure supplement 1E, the original image file for the western blot with HNRNPC (green) and tubulin (red) antibodies on HTD114 cells with and without shRNA against HNRNPC. Figure 3-figure supplement 1E (labels), the same image as above showing labels for the location of HNRNPC and tubulin, and the region of the image used is highlighted in white. Molecular weight standards are shown in red. [file elife-95898-fig3-figsupp1-data1.zip › Figure 3-figure supplement 1 source file data/Figure 3-figure supplement 1 L(labels).tif]

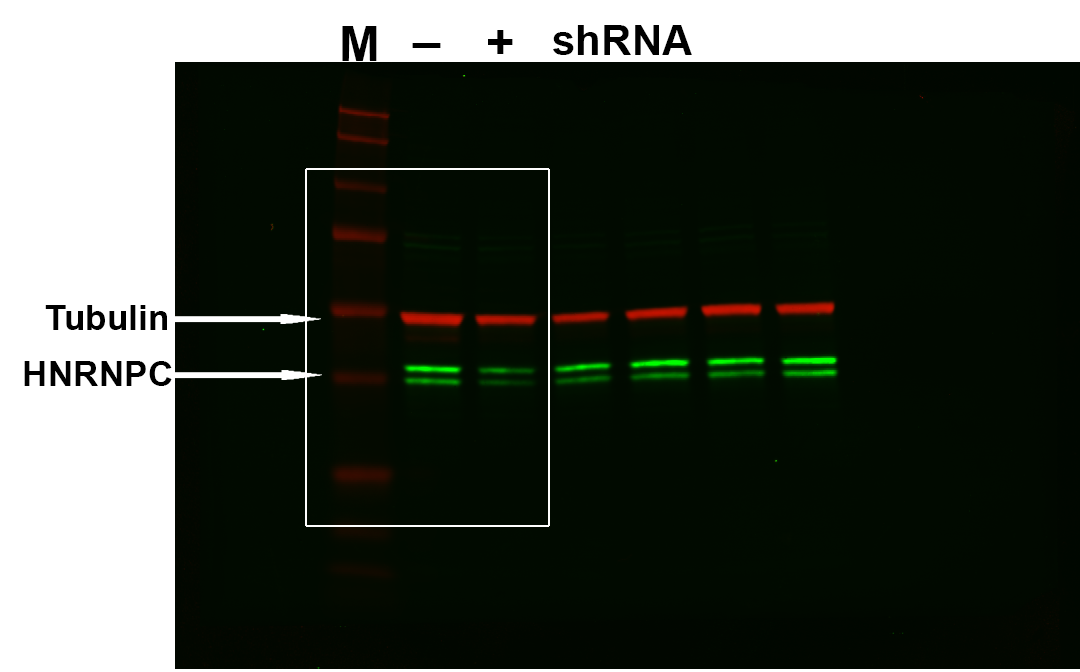

Supplement: Figure 3—figure supplement 1—source data 1. — Figure 3-figure supplement 1A, the original image file for the western blot with HNRNPU (green) and tubulin (red) antibodies on K562 cells with and without shRNA against HNRNPU. Figure 3-figure supplement 1A (labels), the same image as above showing labels for the location of HNRNPU and tubulin, and the region of the image used is highlighted in white. Molecular weight standards are shown in red. Figure 3-figure supplement 1B, the original image file for the western blot with HNRNPU (green) and tubulin (red) antibodies on HTD114 cells with and without shRNA against HNRNPU. Figure 3-figure supplement 1B (labels), the same image as above showing labels for the location of HNRNPU and tubulin, and the region of the image used is highlighted in white. Molecular weight standards are shown in red. Figure 3—figure supplement 1C, the original image file for the western blot with UCHL5 (green) and tubulin (red) antibodies on K562 cells with and without shRNA against UCHL5. Figure 3-figure supplement 1C (labels), the same image as above showing labels for the location of UCHL5 and tubulin, and the region of the image used is highlighted in white. Molecular weight standards are shown in red. Figure 3-figure supplement 1D, the original image file for the western blot with HNRNPC (green) and tubulin (red) antibodies on K562 cells with and without shRNA against HNRNPC. Figure 3-figure supplement 1C (labels), the same image as above showing labels for the location of HNRNPC and tubulin, and the region of the image used is highlighted in white. Molecular weight standards are shown in red. Figure 3—figure supplement 1E, the original image file for the western blot with HNRNPC (green) and tubulin (red) antibodies on HTD114 cells with and without shRNA against HNRNPC. Figure 3-figure supplement 1E (labels), the same image as above showing labels for the location of HNRNPC and tubulin, and the region of the image used is highlighted in white. Molecular weight standards are shown in red. [file elife-95898-fig3-figsupp1-data1.zip › Figure 3-figure supplement 1 source file data/Figure 3-figure supplement 1 E(labels).tif]

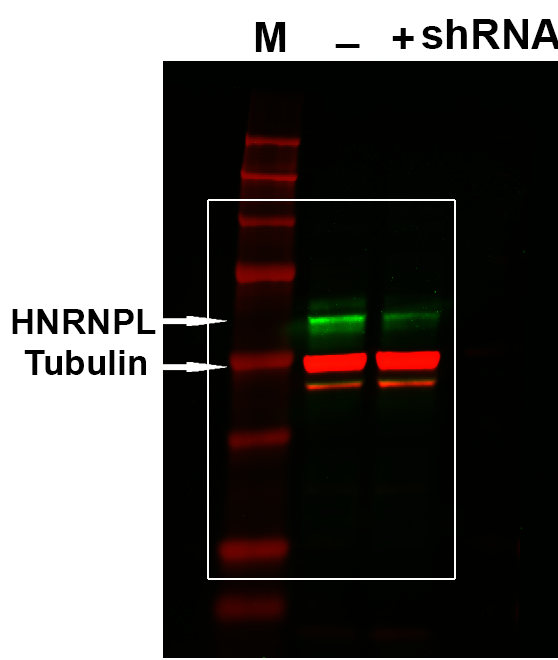

Supplement: Figure 3—figure supplement 1—source data 1. — Figure 3-figure supplement 1A, the original image file for the western blot with HNRNPU (green) and tubulin (red) antibodies on K562 cells with and without shRNA against HNRNPU. Figure 3-figure supplement 1A (labels), the same image as above showing labels for the location of HNRNPU and tubulin, and the region of the image used is highlighted in white. Molecular weight standards are shown in red. Figure 3-figure supplement 1B, the original image file for the western blot with HNRNPU (green) and tubulin (red) antibodies on HTD114 cells with and without shRNA against HNRNPU. Figure 3-figure supplement 1B (labels), the same image as above showing labels for the location of HNRNPU and tubulin, and the region of the image used is highlighted in white. Molecular weight standards are shown in red. Figure 3—figure supplement 1C, the original image file for the western blot with UCHL5 (green) and tubulin (red) antibodies on K562 cells with and without shRNA against UCHL5. Figure 3-figure supplement 1C (labels), the same image as above showing labels for the location of UCHL5 and tubulin, and the region of the image used is highlighted in white. Molecular weight standards are shown in red. Figure 3-figure supplement 1D, the original image file for the western blot with HNRNPC (green) and tubulin (red) antibodies on K562 cells with and without shRNA against HNRNPC. Figure 3-figure supplement 1C (labels), the same image as above showing labels for the location of HNRNPC and tubulin, and the region of the image used is highlighted in white. Molecular weight standards are shown in red. Figure 3—figure supplement 1E, the original image file for the western blot with HNRNPC (green) and tubulin (red) antibodies on HTD114 cells with and without shRNA against HNRNPC. Figure 3-figure supplement 1E (labels), the same image as above showing labels for the location of HNRNPC and tubulin, and the region of the image used is highlighted in white. Molecular weight standards are shown in red. [file elife-95898-fig3-figsupp1-data1.zip › Figure 3-figure supplement 1 source file data/Figure 3-figure supplement 1 I(labels).tif]

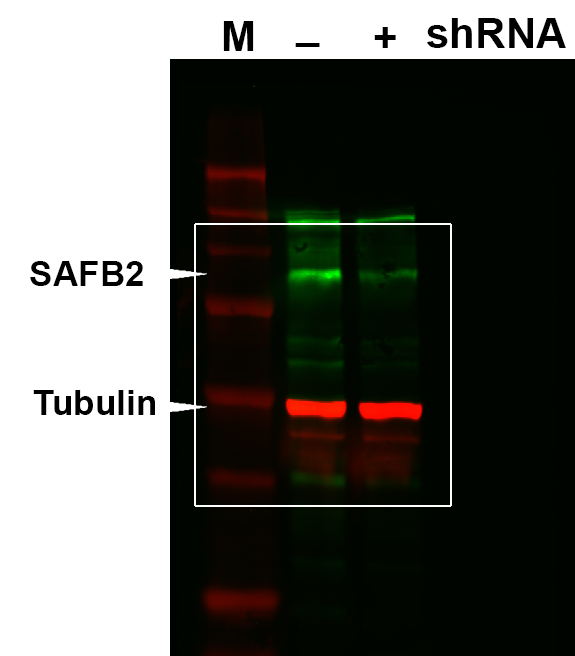

Supplement: Figure 3—figure supplement 1—source data 1. — Figure 3-figure supplement 1A, the original image file for the western blot with HNRNPU (green) and tubulin (red) antibodies on K562 cells with and without shRNA against HNRNPU. Figure 3-figure supplement 1A (labels), the same image as above showing labels for the location of HNRNPU and tubulin, and the region of the image used is highlighted in white. Molecular weight standards are shown in red. Figure 3-figure supplement 1B, the original image file for the western blot with HNRNPU (green) and tubulin (red) antibodies on HTD114 cells with and without shRNA against HNRNPU. Figure 3-figure supplement 1B (labels), the same image as above showing labels for the location of HNRNPU and tubulin, and the region of the image used is highlighted in white. Molecular weight standards are shown in red. Figure 3—figure supplement 1C, the original image file for the western blot with UCHL5 (green) and tubulin (red) antibodies on K562 cells with and without shRNA against UCHL5. Figure 3-figure supplement 1C (labels), the same image as above showing labels for the location of UCHL5 and tubulin, and the region of the image used is highlighted in white. Molecular weight standards are shown in red. Figure 3-figure supplement 1D, the original image file for the western blot with HNRNPC (green) and tubulin (red) antibodies on K562 cells with and without shRNA against HNRNPC. Figure 3-figure supplement 1C (labels), the same image as above showing labels for the location of HNRNPC and tubulin, and the region of the image used is highlighted in white. Molecular weight standards are shown in red. Figure 3—figure supplement 1E, the original image file for the western blot with HNRNPC (green) and tubulin (red) antibodies on HTD114 cells with and without shRNA against HNRNPC. Figure 3-figure supplement 1E (labels), the same image as above showing labels for the location of HNRNPC and tubulin, and the region of the image used is highlighted in white. Molecular weight standards are shown in red. [file elife-95898-fig3-figsupp1-data1.zip › Figure 3-figure supplement 1 source file data/Figure 3-figure supplement 1 N(labels).tif]

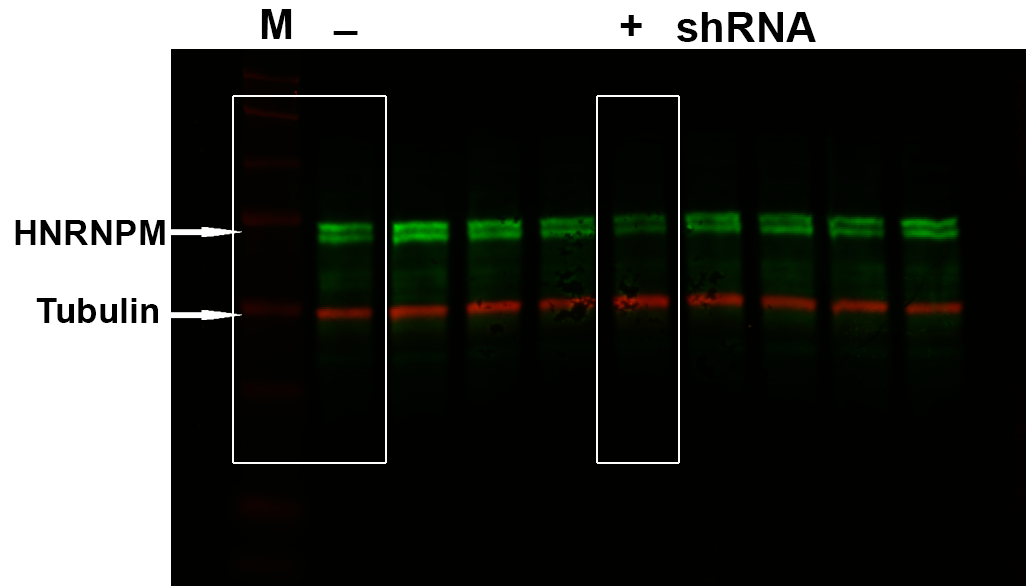

Supplement: Figure 3—figure supplement 1—source data 1. — Figure 3-figure supplement 1A, the original image file for the western blot with HNRNPU (green) and tubulin (red) antibodies on K562 cells with and without shRNA against HNRNPU. Figure 3-figure supplement 1A (labels), the same image as above showing labels for the location of HNRNPU and tubulin, and the region of the image used is highlighted in white. Molecular weight standards are shown in red. Figure 3-figure supplement 1B, the original image file for the western blot with HNRNPU (green) and tubulin (red) antibodies on HTD114 cells with and without shRNA against HNRNPU. Figure 3-figure supplement 1B (labels), the same image as above showing labels for the location of HNRNPU and tubulin, and the region of the image used is highlighted in white. Molecular weight standards are shown in red. Figure 3—figure supplement 1C, the original image file for the western blot with UCHL5 (green) and tubulin (red) antibodies on K562 cells with and without shRNA against UCHL5. Figure 3-figure supplement 1C (labels), the same image as above showing labels for the location of UCHL5 and tubulin, and the region of the image used is highlighted in white. Molecular weight standards are shown in red. Figure 3-figure supplement 1D, the original image file for the western blot with HNRNPC (green) and tubulin (red) antibodies on K562 cells with and without shRNA against HNRNPC. Figure 3-figure supplement 1C (labels), the same image as above showing labels for the location of HNRNPC and tubulin, and the region of the image used is highlighted in white. Molecular weight standards are shown in red. Figure 3—figure supplement 1E, the original image file for the western blot with HNRNPC (green) and tubulin (red) antibodies on HTD114 cells with and without shRNA against HNRNPC. Figure 3-figure supplement 1E (labels), the same image as above showing labels for the location of HNRNPC and tubulin, and the region of the image used is highlighted in white. Molecular weight standards are shown in red. [file elife-95898-fig3-figsupp1-data1.zip › Figure 3-figure supplement 1 source file data/Figure 3-figure supplement 1 G(labels).tif]

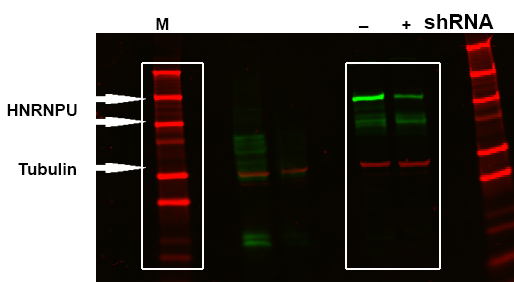

Supplement: Figure 3—figure supplement 1—source data 1. — Figure 3-figure supplement 1A, the original image file for the western blot with HNRNPU (green) and tubulin (red) antibodies on K562 cells with and without shRNA against HNRNPU. Figure 3-figure supplement 1A (labels), the same image as above showing labels for the location of HNRNPU and tubulin, and the region of the image used is highlighted in white. Molecular weight standards are shown in red. Figure 3-figure supplement 1B, the original image file for the western blot with HNRNPU (green) and tubulin (red) antibodies on HTD114 cells with and without shRNA against HNRNPU. Figure 3-figure supplement 1B (labels), the same image as above showing labels for the location of HNRNPU and tubulin, and the region of the image used is highlighted in white. Molecular weight standards are shown in red. Figure 3—figure supplement 1C, the original image file for the western blot with UCHL5 (green) and tubulin (red) antibodies on K562 cells with and without shRNA against UCHL5. Figure 3-figure supplement 1C (labels), the same image as above showing labels for the location of UCHL5 and tubulin, and the region of the image used is highlighted in white. Molecular weight standards are shown in red. Figure 3-figure supplement 1D, the original image file for the western blot with HNRNPC (green) and tubulin (red) antibodies on K562 cells with and without shRNA against HNRNPC. Figure 3-figure supplement 1C (labels), the same image as above showing labels for the location of HNRNPC and tubulin, and the region of the image used is highlighted in white. Molecular weight standards are shown in red. Figure 3—figure supplement 1E, the original image file for the western blot with HNRNPC (green) and tubulin (red) antibodies on HTD114 cells with and without shRNA against HNRNPC. Figure 3-figure supplement 1E (labels), the same image as above showing labels for the location of HNRNPC and tubulin, and the region of the image used is highlighted in white. Molecular weight standards are shown in red. [file elife-95898-fig3-figsupp1-data1.zip › Figure 3-figure supplement 1 source file data/Figure 3-figure supplement 1 B(labels).tif]

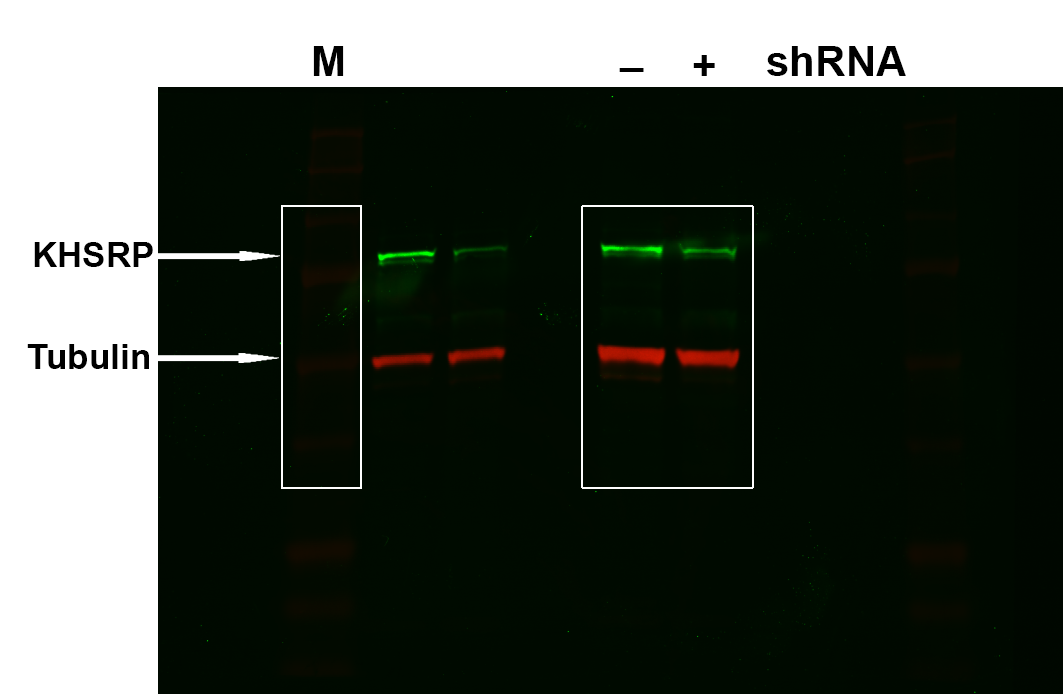

Supplement: Figure 3—figure supplement 1—source data 1. — Figure 3-figure supplement 1A, the original image file for the western blot with HNRNPU (green) and tubulin (red) antibodies on K562 cells with and without shRNA against HNRNPU. Figure 3-figure supplement 1A (labels), the same image as above showing labels for the location of HNRNPU and tubulin, and the region of the image used is highlighted in white. Molecular weight standards are shown in red. Figure 3-figure supplement 1B, the original image file for the western blot with HNRNPU (green) and tubulin (red) antibodies on HTD114 cells with and without shRNA against HNRNPU. Figure 3-figure supplement 1B (labels), the same image as above showing labels for the location of HNRNPU and tubulin, and the region of the image used is highlighted in white. Molecular weight standards are shown in red. Figure 3—figure supplement 1C, the original image file for the western blot with UCHL5 (green) and tubulin (red) antibodies on K562 cells with and without shRNA against UCHL5. Figure 3-figure supplement 1C (labels), the same image as above showing labels for the location of UCHL5 and tubulin, and the region of the image used is highlighted in white. Molecular weight standards are shown in red. Figure 3-figure supplement 1D, the original image file for the western blot with HNRNPC (green) and tubulin (red) antibodies on K562 cells with and without shRNA against HNRNPC. Figure 3-figure supplement 1C (labels), the same image as above showing labels for the location of HNRNPC and tubulin, and the region of the image used is highlighted in white. Molecular weight standards are shown in red. Figure 3—figure supplement 1E, the original image file for the western blot with HNRNPC (green) and tubulin (red) antibodies on HTD114 cells with and without shRNA against HNRNPC. Figure 3-figure supplement 1E (labels), the same image as above showing labels for the location of HNRNPC and tubulin, and the region of the image used is highlighted in white. Molecular weight standards are shown in red. [file elife-95898-fig3-figsupp1-data1.zip › Figure 3-figure supplement 1 source file data/Figure 3-figure supplement 1 K(labels).tif]

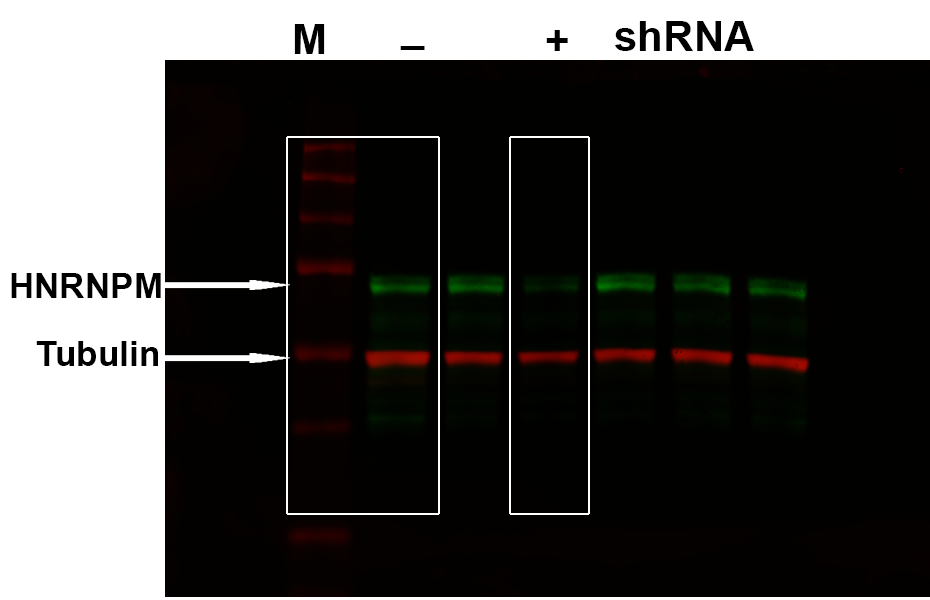

Supplement: Figure 3—figure supplement 1—source data 1. — Figure 3-figure supplement 1A, the original image file for the western blot with HNRNPU (green) and tubulin (red) antibodies on K562 cells with and without shRNA against HNRNPU. Figure 3-figure supplement 1A (labels), the same image as above showing labels for the location of HNRNPU and tubulin, and the region of the image used is highlighted in white. Molecular weight standards are shown in red. Figure 3-figure supplement 1B, the original image file for the western blot with HNRNPU (green) and tubulin (red) antibodies on HTD114 cells with and without shRNA against HNRNPU. Figure 3-figure supplement 1B (labels), the same image as above showing labels for the location of HNRNPU and tubulin, and the region of the image used is highlighted in white. Molecular weight standards are shown in red. Figure 3—figure supplement 1C, the original image file for the western blot with UCHL5 (green) and tubulin (red) antibodies on K562 cells with and without shRNA against UCHL5. Figure 3-figure supplement 1C (labels), the same image as above showing labels for the location of UCHL5 and tubulin, and the region of the image used is highlighted in white. Molecular weight standards are shown in red. Figure 3-figure supplement 1D, the original image file for the western blot with HNRNPC (green) and tubulin (red) antibodies on K562 cells with and without shRNA against HNRNPC. Figure 3-figure supplement 1C (labels), the same image as above showing labels for the location of HNRNPC and tubulin, and the region of the image used is highlighted in white. Molecular weight standards are shown in red. Figure 3—figure supplement 1E, the original image file for the western blot with HNRNPC (green) and tubulin (red) antibodies on HTD114 cells with and without shRNA against HNRNPC. Figure 3-figure supplement 1E (labels), the same image as above showing labels for the location of HNRNPC and tubulin, and the region of the image used is highlighted in white. Molecular weight standards are shown in red. [file elife-95898-fig3-figsupp1-data1.zip › Figure 3-figure supplement 1 source file data/Figure 3-figure supplement 1 H(labels).tif]

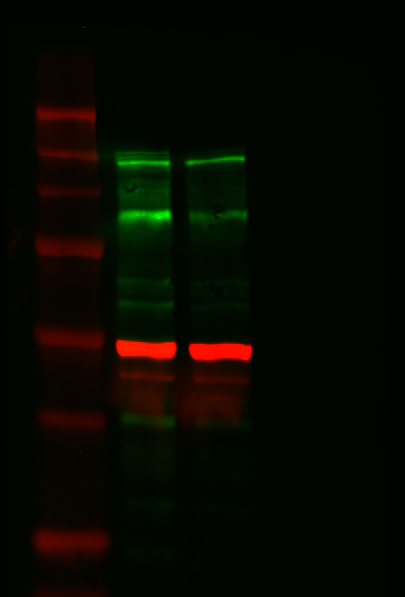

Supplement: Figure 3—figure supplement 1—source data 1. — Figure 3-figure supplement 1A, the original image file for the western blot with HNRNPU (green) and tubulin (red) antibodies on K562 cells with and without shRNA against HNRNPU. Figure 3-figure supplement 1A (labels), the same image as above showing labels for the location of HNRNPU and tubulin, and the region of the image used is highlighted in white. Molecular weight standards are shown in red. Figure 3-figure supplement 1B, the original image file for the western blot with HNRNPU (green) and tubulin (red) antibodies on HTD114 cells with and without shRNA against HNRNPU. Figure 3-figure supplement 1B (labels), the same image as above showing labels for the location of HNRNPU and tubulin, and the region of the image used is highlighted in white. Molecular weight standards are shown in red. Figure 3—figure supplement 1C, the original image file for the western blot with UCHL5 (green) and tubulin (red) antibodies on K562 cells with and without shRNA against UCHL5. Figure 3-figure supplement 1C (labels), the same image as above showing labels for the location of UCHL5 and tubulin, and the region of the image used is highlighted in white. Molecular weight standards are shown in red. Figure 3-figure supplement 1D, the original image file for the western blot with HNRNPC (green) and tubulin (red) antibodies on K562 cells with and without shRNA against HNRNPC. Figure 3-figure supplement 1C (labels), the same image as above showing labels for the location of HNRNPC and tubulin, and the region of the image used is highlighted in white. Molecular weight standards are shown in red. Figure 3—figure supplement 1E, the original image file for the western blot with HNRNPC (green) and tubulin (red) antibodies on HTD114 cells with and without shRNA against HNRNPC. Figure 3-figure supplement 1E (labels), the same image as above showing labels for the location of HNRNPC and tubulin, and the region of the image used is highlighted in white. Molecular weight standards are shown in red. [file elife-95898-fig3-figsupp1-data1.zip › Figure 3-figure supplement 1 source file data/Figure 3-figure supplement 1 N.tif]

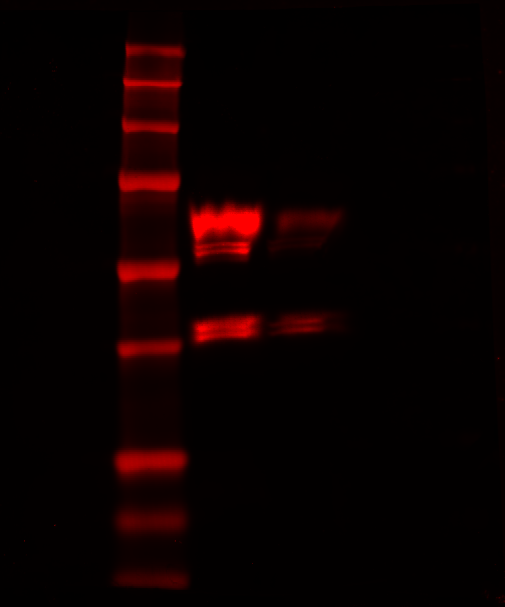

Supplement: Figure 3—figure supplement 1—source data 1. — Figure 3-figure supplement 1A, the original image file for the western blot with HNRNPU (green) and tubulin (red) antibodies on K562 cells with and without shRNA against HNRNPU. Figure 3-figure supplement 1A (labels), the same image as above showing labels for the location of HNRNPU and tubulin, and the region of the image used is highlighted in white. Molecular weight standards are shown in red. Figure 3-figure supplement 1B, the original image file for the western blot with HNRNPU (green) and tubulin (red) antibodies on HTD114 cells with and without shRNA against HNRNPU. Figure 3-figure supplement 1B (labels), the same image as above showing labels for the location of HNRNPU and tubulin, and the region of the image used is highlighted in white. Molecular weight standards are shown in red. Figure 3—figure supplement 1C, the original image file for the western blot with UCHL5 (green) and tubulin (red) antibodies on K562 cells with and without shRNA against UCHL5. Figure 3-figure supplement 1C (labels), the same image as above showing labels for the location of UCHL5 and tubulin, and the region of the image used is highlighted in white. Molecular weight standards are shown in red. Figure 3-figure supplement 1D, the original image file for the western blot with HNRNPC (green) and tubulin (red) antibodies on K562 cells with and without shRNA against HNRNPC. Figure 3-figure supplement 1C (labels), the same image as above showing labels for the location of HNRNPC and tubulin, and the region of the image used is highlighted in white. Molecular weight standards are shown in red. Figure 3—figure supplement 1E, the original image file for the western blot with HNRNPC (green) and tubulin (red) antibodies on HTD114 cells with and without shRNA against HNRNPC. Figure 3-figure supplement 1E (labels), the same image as above showing labels for the location of HNRNPC and tubulin, and the region of the image used is highlighted in white. Molecular weight standards are shown in red. [file elife-95898-fig3-figsupp1-data1.zip › Figure 3-figure supplement 1 source file data/Figure 3-figure supplement 1 O.tif]

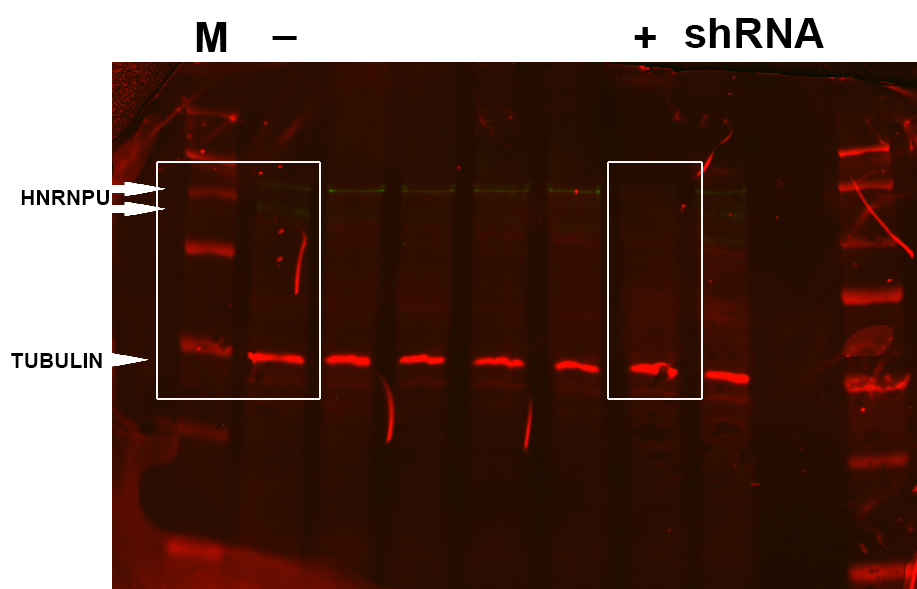

Supplement: Figure 3—figure supplement 1—source data 1. — Figure 3-figure supplement 1A, the original image file for the western blot with HNRNPU (green) and tubulin (red) antibodies on K562 cells with and without shRNA against HNRNPU. Figure 3-figure supplement 1A (labels), the same image as above showing labels for the location of HNRNPU and tubulin, and the region of the image used is highlighted in white. Molecular weight standards are shown in red. Figure 3-figure supplement 1B, the original image file for the western blot with HNRNPU (green) and tubulin (red) antibodies on HTD114 cells with and without shRNA against HNRNPU. Figure 3-figure supplement 1B (labels), the same image as above showing labels for the location of HNRNPU and tubulin, and the region of the image used is highlighted in white. Molecular weight standards are shown in red. Figure 3—figure supplement 1C, the original image file for the western blot with UCHL5 (green) and tubulin (red) antibodies on K562 cells with and without shRNA against UCHL5. Figure 3-figure supplement 1C (labels), the same image as above showing labels for the location of UCHL5 and tubulin, and the region of the image used is highlighted in white. Molecular weight standards are shown in red. Figure 3-figure supplement 1D, the original image file for the western blot with HNRNPC (green) and tubulin (red) antibodies on K562 cells with and without shRNA against HNRNPC. Figure 3-figure supplement 1C (labels), the same image as above showing labels for the location of HNRNPC and tubulin, and the region of the image used is highlighted in white. Molecular weight standards are shown in red. Figure 3—figure supplement 1E, the original image file for the western blot with HNRNPC (green) and tubulin (red) antibodies on HTD114 cells with and without shRNA against HNRNPC. Figure 3-figure supplement 1E (labels), the same image as above showing labels for the location of HNRNPC and tubulin, and the region of the image used is highlighted in white. Molecular weight standards are shown in red. [file elife-95898-fig3-figsupp1-data1.zip › Figure 3-figure supplement 1 source file data/Figure 3-figure supplement 1 A(labels).tif]

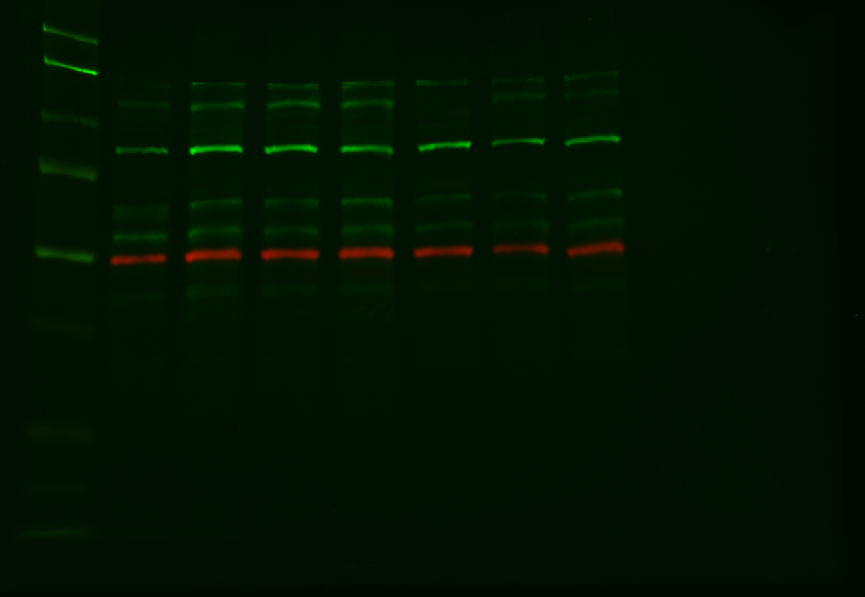

Supplement: Figure 3—figure supplement 1—source data 1. — Figure 3-figure supplement 1A, the original image file for the western blot with HNRNPU (green) and tubulin (red) antibodies on K562 cells with and without shRNA against HNRNPU. Figure 3-figure supplement 1A (labels), the same image as above showing labels for the location of HNRNPU and tubulin, and the region of the image used is highlighted in white. Molecular weight standards are shown in red. Figure 3-figure supplement 1B, the original image file for the western blot with HNRNPU (green) and tubulin (red) antibodies on HTD114 cells with and without shRNA against HNRNPU. Figure 3-figure supplement 1B (labels), the same image as above showing labels for the location of HNRNPU and tubulin, and the region of the image used is highlighted in white. Molecular weight standards are shown in red. Figure 3—figure supplement 1C, the original image file for the western blot with UCHL5 (green) and tubulin (red) antibodies on K562 cells with and without shRNA against UCHL5. Figure 3-figure supplement 1C (labels), the same image as above showing labels for the location of UCHL5 and tubulin, and the region of the image used is highlighted in white. Molecular weight standards are shown in red. Figure 3-figure supplement 1D, the original image file for the western blot with HNRNPC (green) and tubulin (red) antibodies on K562 cells with and without shRNA against HNRNPC. Figure 3-figure supplement 1C (labels), the same image as above showing labels for the location of HNRNPC and tubulin, and the region of the image used is highlighted in white. Molecular weight standards are shown in red. Figure 3—figure supplement 1E, the original image file for the western blot with HNRNPC (green) and tubulin (red) antibodies on HTD114 cells with and without shRNA against HNRNPC. Figure 3-figure supplement 1E (labels), the same image as above showing labels for the location of HNRNPC and tubulin, and the region of the image used is highlighted in white. Molecular weight standards are shown in red. [file elife-95898-fig3-figsupp1-data1.zip › Figure 3-figure supplement 1 source file data/Figure 3-figure supplement 1 M.tif]

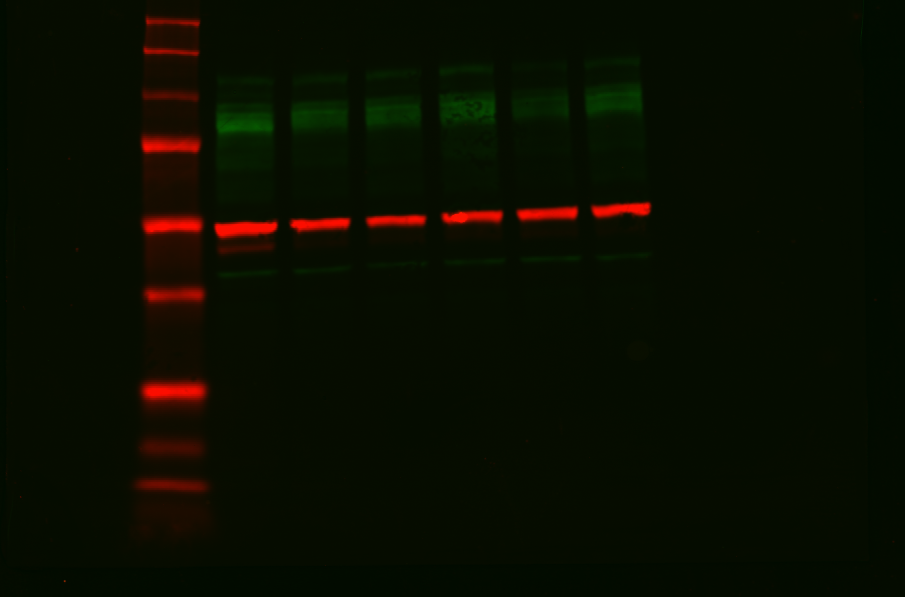

Supplement: Figure 3—figure supplement 1—source data 1. — Figure 3-figure supplement 1A, the original image file for the western blot with HNRNPU (green) and tubulin (red) antibodies on K562 cells with and without shRNA against HNRNPU. Figure 3-figure supplement 1A (labels), the same image as above showing labels for the location of HNRNPU and tubulin, and the region of the image used is highlighted in white. Molecular weight standards are shown in red. Figure 3-figure supplement 1B, the original image file for the western blot with HNRNPU (green) and tubulin (red) antibodies on HTD114 cells with and without shRNA against HNRNPU. Figure 3-figure supplement 1B (labels), the same image as above showing labels for the location of HNRNPU and tubulin, and the region of the image used is highlighted in white. Molecular weight standards are shown in red. Figure 3—figure supplement 1C, the original image file for the western blot with UCHL5 (green) and tubulin (red) antibodies on K562 cells with and without shRNA against UCHL5. Figure 3-figure supplement 1C (labels), the same image as above showing labels for the location of UCHL5 and tubulin, and the region of the image used is highlighted in white. Molecular weight standards are shown in red. Figure 3-figure supplement 1D, the original image file for the western blot with HNRNPC (green) and tubulin (red) antibodies on K562 cells with and without shRNA against HNRNPC. Figure 3-figure supplement 1C (labels), the same image as above showing labels for the location of HNRNPC and tubulin, and the region of the image used is highlighted in white. Molecular weight standards are shown in red. Figure 3—figure supplement 1E, the original image file for the western blot with HNRNPC (green) and tubulin (red) antibodies on HTD114 cells with and without shRNA against HNRNPC. Figure 3-figure supplement 1E (labels), the same image as above showing labels for the location of HNRNPC and tubulin, and the region of the image used is highlighted in white. Molecular weight standards are shown in red. [file elife-95898-fig3-figsupp1-data1.zip › Figure 3-figure supplement 1 source file data/Figure 3-figure supplement 1 L.tif]

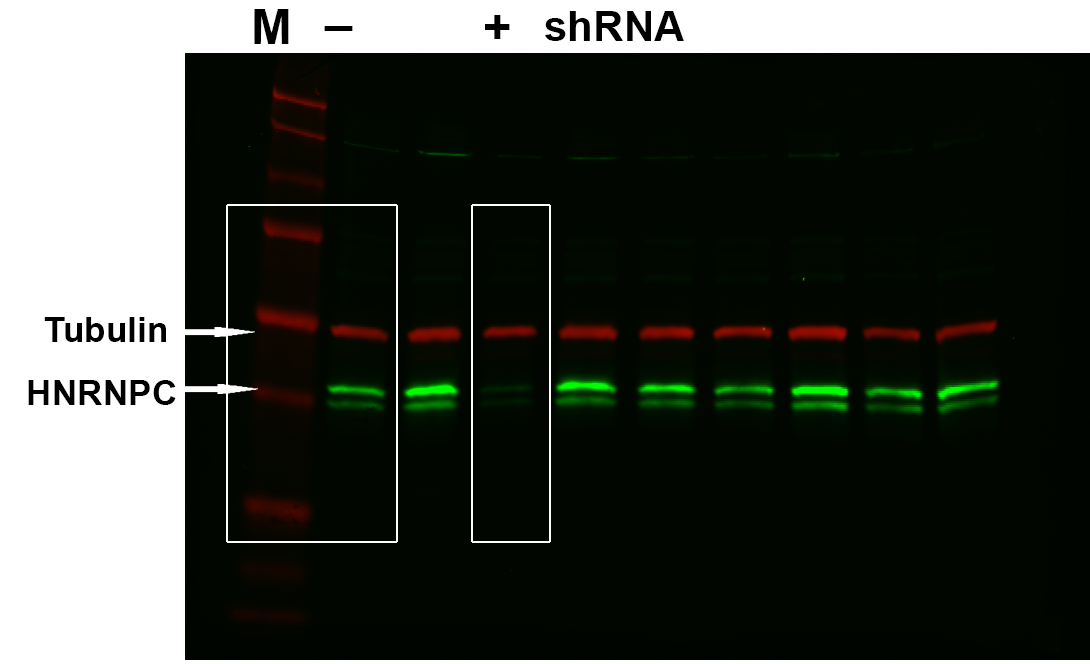

Supplement: Figure 3—figure supplement 1—source data 1. — Figure 3-figure supplement 1A, the original image file for the western blot with HNRNPU (green) and tubulin (red) antibodies on K562 cells with and without shRNA against HNRNPU. Figure 3-figure supplement 1A (labels), the same image as above showing labels for the location of HNRNPU and tubulin, and the region of the image used is highlighted in white. Molecular weight standards are shown in red. Figure 3-figure supplement 1B, the original image file for the western blot with HNRNPU (green) and tubulin (red) antibodies on HTD114 cells with and without shRNA against HNRNPU. Figure 3-figure supplement 1B (labels), the same image as above showing labels for the location of HNRNPU and tubulin, and the region of the image used is highlighted in white. Molecular weight standards are shown in red. Figure 3—figure supplement 1C, the original image file for the western blot with UCHL5 (green) and tubulin (red) antibodies on K562 cells with and without shRNA against UCHL5. Figure 3-figure supplement 1C (labels), the same image as above showing labels for the location of UCHL5 and tubulin, and the region of the image used is highlighted in white. Molecular weight standards are shown in red. Figure 3-figure supplement 1D, the original image file for the western blot with HNRNPC (green) and tubulin (red) antibodies on K562 cells with and without shRNA against HNRNPC. Figure 3-figure supplement 1C (labels), the same image as above showing labels for the location of HNRNPC and tubulin, and the region of the image used is highlighted in white. Molecular weight standards are shown in red. Figure 3—figure supplement 1E, the original image file for the western blot with HNRNPC (green) and tubulin (red) antibodies on HTD114 cells with and without shRNA against HNRNPC. Figure 3-figure supplement 1E (labels), the same image as above showing labels for the location of HNRNPC and tubulin, and the region of the image used is highlighted in white. Molecular weight standards are shown in red. [file elife-95898-fig3-figsupp1-data1.zip › Figure 3-figure supplement 1 source file data/Figure 3-figure supplement 1 D(labels).tif]

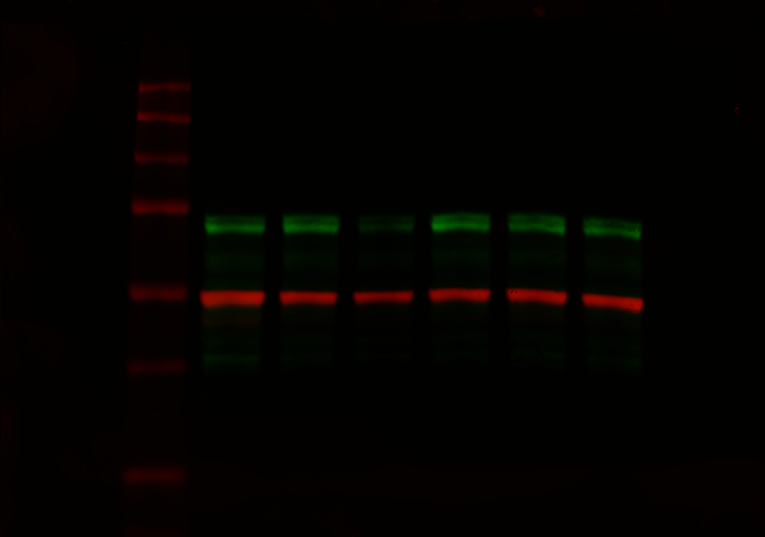

Supplement: Figure 3—figure supplement 1—source data 1. — Figure 3-figure supplement 1A, the original image file for the western blot with HNRNPU (green) and tubulin (red) antibodies on K562 cells with and without shRNA against HNRNPU. Figure 3-figure supplement 1A (labels), the same image as above showing labels for the location of HNRNPU and tubulin, and the region of the image used is highlighted in white. Molecular weight standards are shown in red. Figure 3-figure supplement 1B, the original image file for the western blot with HNRNPU (green) and tubulin (red) antibodies on HTD114 cells with and without shRNA against HNRNPU. Figure 3-figure supplement 1B (labels), the same image as above showing labels for the location of HNRNPU and tubulin, and the region of the image used is highlighted in white. Molecular weight standards are shown in red. Figure 3—figure supplement 1C, the original image file for the western blot with UCHL5 (green) and tubulin (red) antibodies on K562 cells with and without shRNA against UCHL5. Figure 3-figure supplement 1C (labels), the same image as above showing labels for the location of UCHL5 and tubulin, and the region of the image used is highlighted in white. Molecular weight standards are shown in red. Figure 3-figure supplement 1D, the original image file for the western blot with HNRNPC (green) and tubulin (red) antibodies on K562 cells with and without shRNA against HNRNPC. Figure 3-figure supplement 1C (labels), the same image as above showing labels for the location of HNRNPC and tubulin, and the region of the image used is highlighted in white. Molecular weight standards are shown in red. Figure 3—figure supplement 1E, the original image file for the western blot with HNRNPC (green) and tubulin (red) antibodies on HTD114 cells with and without shRNA against HNRNPC. Figure 3-figure supplement 1E (labels), the same image as above showing labels for the location of HNRNPC and tubulin, and the region of the image used is highlighted in white. Molecular weight standards are shown in red. [file elife-95898-fig3-figsupp1-data1.zip › Figure 3-figure supplement 1 source file data/Figure 3-figure supplement 1 H.tif]

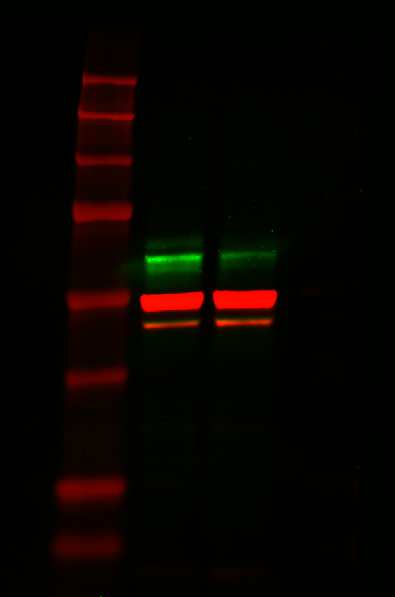

Supplement: Figure 3—figure supplement 1—source data 1. — Figure 3-figure supplement 1A, the original image file for the western blot with HNRNPU (green) and tubulin (red) antibodies on K562 cells with and without shRNA against HNRNPU. Figure 3-figure supplement 1A (labels), the same image as above showing labels for the location of HNRNPU and tubulin, and the region of the image used is highlighted in white. Molecular weight standards are shown in red. Figure 3-figure supplement 1B, the original image file for the western blot with HNRNPU (green) and tubulin (red) antibodies on HTD114 cells with and without shRNA against HNRNPU. Figure 3-figure supplement 1B (labels), the same image as above showing labels for the location of HNRNPU and tubulin, and the region of the image used is highlighted in white. Molecular weight standards are shown in red. Figure 3—figure supplement 1C, the original image file for the western blot with UCHL5 (green) and tubulin (red) antibodies on K562 cells with and without shRNA against UCHL5. Figure 3-figure supplement 1C (labels), the same image as above showing labels for the location of UCHL5 and tubulin, and the region of the image used is highlighted in white. Molecular weight standards are shown in red. Figure 3-figure supplement 1D, the original image file for the western blot with HNRNPC (green) and tubulin (red) antibodies on K562 cells with and without shRNA against HNRNPC. Figure 3-figure supplement 1C (labels), the same image as above showing labels for the location of HNRNPC and tubulin, and the region of the image used is highlighted in white. Molecular weight standards are shown in red. Figure 3—figure supplement 1E, the original image file for the western blot with HNRNPC (green) and tubulin (red) antibodies on HTD114 cells with and without shRNA against HNRNPC. Figure 3-figure supplement 1E (labels), the same image as above showing labels for the location of HNRNPC and tubulin, and the region of the image used is highlighted in white. Molecular weight standards are shown in red. [file elife-95898-fig3-figsupp1-data1.zip › Figure 3-figure supplement 1 source file data/Figure 3-figure supplement 1 I.tif]

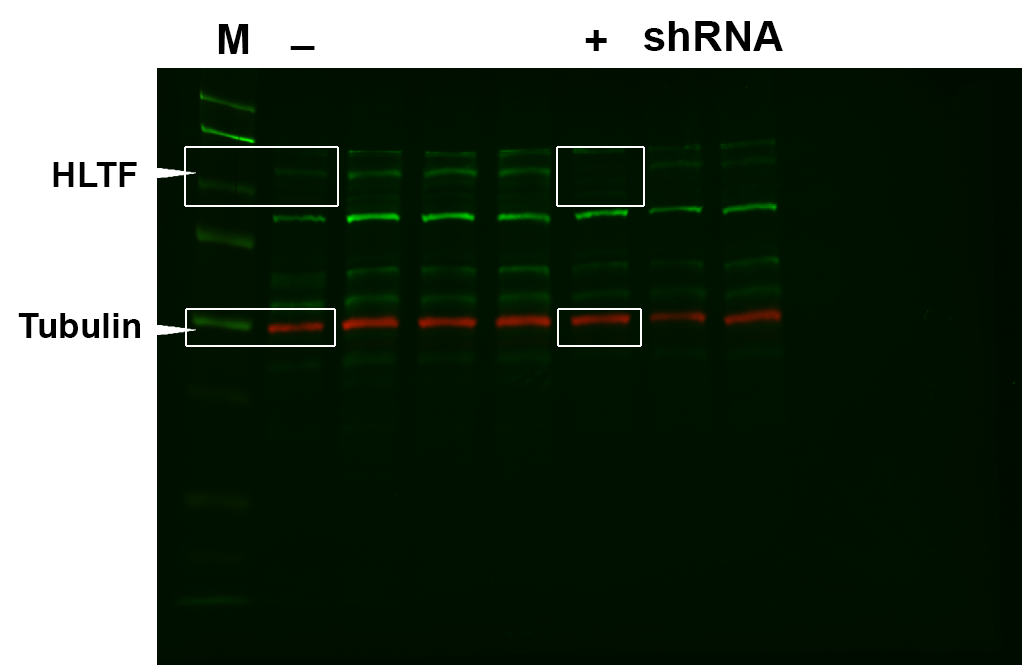

Supplement: Figure 3—figure supplement 1—source data 1. — Figure 3-figure supplement 1A, the original image file for the western blot with HNRNPU (green) and tubulin (red) antibodies on K562 cells with and without shRNA against HNRNPU. Figure 3-figure supplement 1A (labels), the same image as above showing labels for the location of HNRNPU and tubulin, and the region of the image used is highlighted in white. Molecular weight standards are shown in red. Figure 3-figure supplement 1B, the original image file for the western blot with HNRNPU (green) and tubulin (red) antibodies on HTD114 cells with and without shRNA against HNRNPU. Figure 3-figure supplement 1B (labels), the same image as above showing labels for the location of HNRNPU and tubulin, and the region of the image used is highlighted in white. Molecular weight standards are shown in red. Figure 3—figure supplement 1C, the original image file for the western blot with UCHL5 (green) and tubulin (red) antibodies on K562 cells with and without shRNA against UCHL5. Figure 3-figure supplement 1C (labels), the same image as above showing labels for the location of UCHL5 and tubulin, and the region of the image used is highlighted in white. Molecular weight standards are shown in red. Figure 3-figure supplement 1D, the original image file for the western blot with HNRNPC (green) and tubulin (red) antibodies on K562 cells with and without shRNA against HNRNPC. Figure 3-figure supplement 1C (labels), the same image as above showing labels for the location of HNRNPC and tubulin, and the region of the image used is highlighted in white. Molecular weight standards are shown in red. Figure 3—figure supplement 1E, the original image file for the western blot with HNRNPC (green) and tubulin (red) antibodies on HTD114 cells with and without shRNA against HNRNPC. Figure 3-figure supplement 1E (labels), the same image as above showing labels for the location of HNRNPC and tubulin, and the region of the image used is highlighted in white. Molecular weight standards are shown in red. [file elife-95898-fig3-figsupp1-data1.zip › Figure 3-figure supplement 1 source file data/Figure 3-figure supplement 1 M(Labels).tif]
